# Exploring Emerging Therapeutic Targets in Osteosarcoma by Revisiting the Immune and Cancer-Intrinsic Hallmarks of Cancer

**DOI:** 10.3390/cancers17233846

**Published:** 2025-11-30

**Authors:** Lidia Tarone, Antonella Iacoviello, Antonino Di Lorenzo, Roberta Verta, Chiara Cossu, Laura Conti, Federica Cavallo, Federica Riccardo

**Affiliations:** Laboratory of OncoImmunology, Molecular Biotechnology Center “Guido Tarone”, Department of Molecular Biotechnology and Health Sciences, University of Turin, 10126 Turin, Italy; lidia.tarone@unito.it (L.T.); antonella.iacoviello@unito.it (A.I.); antonino.dilorenzo@unito.it (A.D.L.); roberta.verta@unito.it (R.V.); chiara.cossu@unito.it (C.C.); laura.conti@unito.it (L.C.)

**Keywords:** osteosarcoma, CSPG4, xCT, TLR2, cancer hallmarks, immunotherapy, combinatorial strategies

## Abstract

Osteosarcoma (OSA) is an aggressive primary bone cancer mainly affecting the pediatric population. Despite intensive multimodal treatments, therapeutic progress has remained limited for decades, resulting in high recurrence rates, poor prognosis driven by metastatic progression, and severe chemotherapy-associated toxicities. To advance the development of more effective and safer therapeutic strategies, our recent studies identified Chondroitin Sulfate Proteoglycan (CSPG)4 as a relevant mediator of the malignant behavior of OSA cells. Targeting CSPG4 DNA-based vaccine demonstrated encouraging antitumor activity against OSA. Nevertheless, since single-agent immunotherapies are often constrained by tumor immune escape, the need for rational combinatorial strategies is of utmost importance. In this perspective, we broaden our analysis to include other potentially complementary targets beyond CSPG4, which may contribute to OSA pathogenesis. Among these, the cystine/glutamate antiporter xCT and Toll-like Receptor 2 (TLR2) emerge as particularly promising due to their established role in tumor progression, therapy resistance, and immune modulation. We discuss the contribution of all these molecules in major hallmarks of OSA—(1) proliferative and survival advantages, (2) metastasis and angiogenesis, and (3) immune evasion—and examine potential strategies for their combined targeting. By leveraging knowledge gained from other cancer models and integrating it with the distinct biological and clinical features of OSA, this perspective seeks to outline rational and innovative combinatorial strategies that may overcome current therapeutic limitations and ultimately improve patient outcomes.

## Simple Summary

Osteosarcoma (OSA) is a rare but aggressive bone cancer that occurs mainly in children and young adults. Its complex biology makes it one of the most challenging tumors to treat. For decades, therapy has relied on intensive combinations of surgery and chemotherapy, often associated with poor outcomes and severe side effects. This highlights the urgent need to identify novel treatments. To uncover new potential OSA vulnerabilities, here we explore the involvement of three appealing players—Chondroitin Sulfate Proteoglycan (CSPG)4, xCT, and Toll-like Receptor 2 (TLR2)—in the main hallmarks of OSA: proliferation and survival, metastasis and angiogenesis, and immune evasion. By understanding how these molecules interact within OSA biology, we envisage new combinatorial therapies to attack the disease from multiple angles. This approach could open new therapeutic avenues for OSA patients and provide broader insights into developing strategies against other still-incurable cancers.

## 1. Introduction

Osteosarcoma (OSA) is a rare tumor of bones, presenting a bimodal age distribution. The first peak is observed in children and young adolescents, while the second peak is in the elderly. A middle lower plateau is observed in people over 65 years [1,2]. OSA is usually defined, however, as a pediatric cancer, accounting for 60% of bone tumors in the childhood population [3]. This tumor most commonly occurs in the metaphysis of long bones, while it rarely arises in the axial skeleton and other sites [4]. Etiological clues have been associated with OSA onset, assuming its occurrence with puberty, height, and disorders of bone growth and remodeling; nonetheless, definitive environmental risk factors have not been identified up to now. Genetic factors like mutations in the TP53 and RB1 genes may contribute to OSA onset and are linked to increased risk [5].

The current standard of care, which includes surgery and neoadjuvant/adjuvant chemotherapy, has not changed since the 1970s, even though several advances have been made during the past decades. Standard multi-drug chemotherapy protocols include the combination of methotrexate (MTX), doxorubicin (DOXO), cisplatin (CDDP), and ifosfamide (IFO), each targeting cancer cells through different mechanisms of action. MTX acts as an antifolate antimetabolite by inhibiting the proliferation of rapidly dividing cells. DOXO belongs to the anthracycline family and intercalates DNA, inhibiting topoisomerase II function and inducing DNA damage, ultimately leading to apoptosis. CDDP is a cytotoxic drug capable of creating cross-links with purines on DNA, hence interfering with DNA synthesis and causing DNA damage [6]. Finally, IFO blocks DNA replication following its metabolization to mafosfamide, thereby preventing cell division [7].

However, the use of these drugs, alone or combined, has limits, including elevated toxicity, which is especially concerning given the young age of patients. Moreover, their efficacy remains insufficient in high-risk cases, with particularly low survival rates among patients presenting with advanced disease [7]. Indeed, while this multi-drug chemotherapy can enhance tumor reduction and promote substantial tumor shrinkage—enabling limb-sparing surgery as a primary option and alternative to full amputation—leading to survival rates of 65–70% in patients with localized tumors [8], outcomes for patients with metastatic disease remain suboptimal. OSA has a high tendency to metastasize, mainly to the lungs, with metastases occurring in approximately 65% of patients [4]. In about 20% of cases, metastatic lesions are already present at the time of diagnosis, dramatically dropping the survival to 20% [4]. Generally, chemotherapy is often ineffective against metastatic dissemination, and patients who fail to respond to treatment rapidly experience progressive disease, which is ultimately incurable and represents the leading cause of OSA-related death. Moreover, even among patients who initially respond to chemotherapy, approximately 25% will still relapse, usually within 3 years [9].

OSA cells are indeed characterized by both intrinsic and acquired mechanisms that undermine chemotherapy efficacy, making drug resistance the key reason for treatment failure and relapse.

Genetic mutations, along with alterations in key signaling pathways, render a subset of OSA cells intrinsically resistant to first-line treatments [10]. In addition, cancer cells that survive initial chemotherapy cycles frequently undergo adaptive reprogramming, leading to acquired resistance to subsequent therapies. Identifying novel therapeutic options for OSA patients is therefore an unmet clinical need.

Targeted therapies—including the insulin-like growth factor receptor (IGF-1R) inhibitors and tyrosine kinase inhibitors such as regorafenib, cabozantinib, sorafenib (with or without everolimus), and pazopanib—as well as the macrophage-activating agent mifamurtide (approved in Europe), and immunotherapies such as checkpoint inhibitors and CAR-T approaches (mainly targeting GD2 and B7-H3), are currently under clinical investigation for OSA. However, to date, none of these strategies have produced substantial improvements in survival for patients with relapsed or refractory disease. This stagnation in clinical outcomes highlights the urgent need for innovative therapeutic approaches and supports the exploration of novel biological targets that can address tumor heterogeneity and mechanisms of resistance. Such strategies may enhance preclinical findings by complementing, rather than replacing, existing treatment modalities.

It is important to emphasize that, unlike many other tumor types, OSA has not experienced meaningful therapeutic advances in recent decades. This has been largely due to limited understanding of actionable targets, the highly immunosuppressive tumor microenvironment (TME), and the lack of effective second-line treatment options.

## 2. Tackling OSA from Multiple Angles

In general, the concept of monotherapy, targeting the tumor from a “single angle”, has limited effectiveness. This is not unexpected in OSA, given its complexity. The identification of targets that contribute to one or more hallmarks of OSA landscape may provide the opportunity to interfere with tumor progression at multiple levels.

In our efforts to discover novel and reliable therapeutic targets in OSA, we have identified Chondroitin Sulfate proteoglycan 4 (CSPG4) as a promising candidate involved in OSA malignancy. CSPG4 is a cell surface proteoglycan implicated in the regulation of several key oncogenic functions and frequently overexpressed in aggressive cancers, including melanoma and sarcomas, where its expression correlates with aggressive disease [11,12,13,14,15,16,17,18]. In the context of OSA, we have recently demonstrated CSPG4 overexpression at mRNA and protein level in human and canine OSA patients, respectively, and a possible clinical implication has been suggested by a correlation between its overexpression and a shorter survival for both OSA-affected humans and dogs [12]. As a step forward, we have recently demonstrated that CSPG4 downmodulation in human OSA cells impairs malignant behavior and enhances sensitivity to chemotherapy [11,12]. Furthermore, CSPG4 targeting through a chimeric human/dog (HuDo)-CSPG4 DNA vaccine has shown promising anti-tumor activity in highly translational comparative OSA models, including human xenograft mouse models and canine patients with spontaneous OSA [19]. While further investigations are underway to better understand the role of CSPG4 in OSA and to evaluate the efficacy of its immune-targeting in combinatorial approaches with the standard of care (personal communication), focusing on a single molecular target carries the risk of tumor escape. Therefore, a multi-targeted approach that attacks the tumor from different angles may represent a successful strategy. We have been investigating specific molecular targets for several years, mainly in other tumor types such as breast cancer. However, considering the functional roles of these molecules and the increasing evidence of their involvement across different cancers, we are now focusing our attention on their potential implication in the OSA setting. In this regard, the cystine/glutamate antiporter xCT, and Toll-like Receptor 2 (TLR2) represent particularly interesting candidates, having a key role in cancer progression and therapy-resistance and being (over)expressed also in OSA [11,12,20,21,22,23,24,25,26].

xCT, the substrate-specific chain of the cystine/glutamate antiporter system xc^-^, plays a crucial role in maintaining redox balance, thereby sustaining tumor survival under stress conditions [27]. Notably, in sarcomas it can be stabilized by IL-1 receptor accessory protein (IL1RAP), increasing its antiporter activity and conferring resistance to anoikis and protection against ferroptosis [28]. Additionally, TLR2, a pattern-recognition receptor (PRR), links tumor biology to the immune system by modulating inflammation and shaping the TME [29]. Therefore, the combined targeting of these molecules may provide complementary and synergistic effects, acting on multiple fronts to counteract OSA progression and improve patient outcomes.

Building on their established intrinsic roles in tumor biology and tumor-stroma interactions, redox balance, and immune modulation, the significance of CSPG4, xCT, and TLR2 in OSA may be further understood in the context of the pervasive TP53 and RB1 alterations that define this tumor type [5,30]. Deregulated oncogenic signaling downstream of TP53/RB1 loss may upregulate CSPG4 or potentiate its pro-tumoral functions, including invasion, proliferation, and stromal recruitment [13]. Specifically, loss of TP53-mediated repression or RB1/E2F dysregulation can increase the expression of platelet-derived growth factor receptor alpha (PDGFRα), a receptor tyrosine kinase that promotes proliferation, angiogenesis, and stromal recruitment and is overexpressed in a significant proportion of OSA cases; CSPG4 has been implicated in potentiating PDGFRα signaling through ligand presentation [31,32].

TP53 perturbation also shifts antioxidant signaling through NRF2 and stress response pathways, creating selective pressure for xCT upregulation to sustain glutathione (GSH) synthesis, suppress ferroptosis, and buffer oxidative stress [27,33]. TLR2 contributes to immune modulation, with RB1 loss fostering a proinflammatory or immunomodulatory milieu that favors its expression. Tumor cells can exploit TLR2–MyD88/NF-κB signaling for survival, proliferation, and immune evasion [29,34]. In addition, TLR2 can activate STAT3 either directly [35] or by inducing the release of cytokines such as IL-6 that induce its activation [36] and can induce NF-κB activation [37] which directly drives transcriptional upregulation of xCT [38]. In this way, TLR2/NF-κB signaling establishes a molecular bridge between inflammatory cues and antioxidant/redox programs, enabling tumor cells to resist ferroptosis and oxidative stress [38,39]. This link positions TLR2 not only as an immune-modulatory receptor but also as an upstream regulator of metabolic survival pathways in cancer, possibly also in OSA.

**Figure 1:**
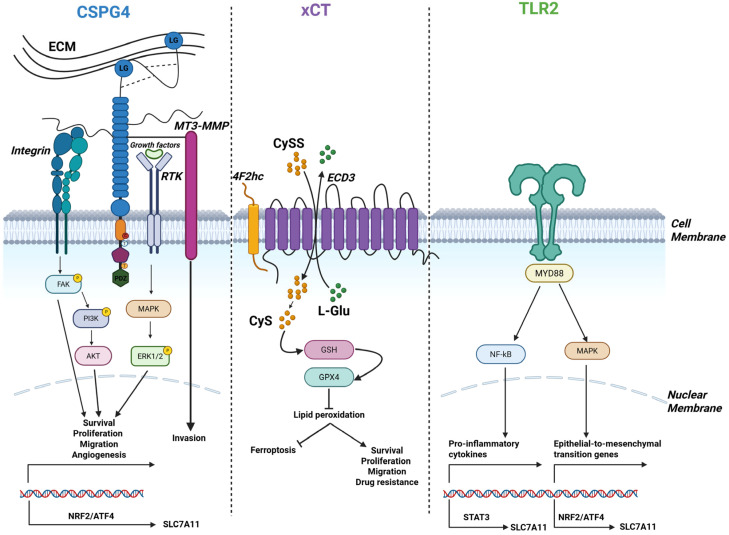
Schematic depiction of CSPG4, xCT, and TLR2 and their associated regulatory signaling pathways. CSPG4, xCT, and TLR2 signaling pathways may converge and regulate transcriptional axes which cooperate to sustain the activation of tumor-supportive processes, thereby directly or indirectly promoting OSA malignant properties. Created in https://BioRender.com.

Consequently, CSPG4, xCT, and TLR2 can represent functional effectors of the TP53/RB1-deficient state in OSA. Mechanistically, overlapping signaling and transcriptional control axes, particularly the PI3K/AKT-mTOR-ATF4/NRF2 pathway, may explain the co-expression of these molecules: CSPG4 and PDGFRα activate PI3K/AKT, xCT is induced downstream via NRF2/ATF4 and STAT3, and TLR2 can further amplify PI3K/AKT, NF-κB, and STAT3 signaling [13,29,40]. CSPG4 also promotes MAPK signaling through interactions with PDGFRα/epidermal growth factor receptor (EGFR) [13,32], while TLR2 can likewise activate ERK/MAPK [29,41]; MAPK activation in turn enhances ATF4/NRF2 activity, providing an additional route toward xCT induction [40,42] (Figure 1).

The hypoxia–HIF1α axis may further reinforce this program, as HIF1α induces xCT in OSA cells [43], enhances CSPG4 expression [44], and upregulates TLR2 in tumor and immune cells [45]. Consequently, in the hypoxic OSA microenvironment, STAT3-mediated signaling may cooperate with HIF1α to maintain high levels of xCT and potentially amplify CSPG4 and TLR2 expression, linking TP53/RB1 deficiency to invasive, stress-resistant, and immunomodulatory tumor phenotypes.

Taken together, these observations position CSPG4, xCT, and TLR2 as a novel, interconnected set of promising biomarkers and putative therapeutic targets in OSA, warranting further mechanistic and translational investigation.

## 3. CSPG4

CSPG4 is a transmembrane proteoglycan, highly expressed in different tumor histotypes [46,47,48,49], while barely present in healthy tissues [50], where its expression is generally restricted to precursor or progenitor cells, whilst absent in terminally differentiated cells [51]. CSPG4 extracellular region is composed of three structural domains (D1-D2-D3) and can be identified either as a core protein characterized by a unique structural complexity, which permits the interaction with extracellular matrix (ECM) components, grow factors and cell-surface receptors, or a chondroitin-sulfate (CS) decorated proteoglycan. CS chains, allow CSPG4 to interact with different molecules, driving the modulation of numerous signaling pathways [15]. The transmembrane region is involved in membrane localization, whereas the cytoplasmic portion, which contains tyrosine phosphoacceptor sites for PKCα and ERK 1,2, hence orchestrating cell proliferation and motility [18]. The inner C-terminus contains a 4 residue PDZ domain-binding motif (PDZ), that is responsible for interactions with various PDZ domain-containing binding partners. The cytoplasmic part of CSPG4 plays the functional role of the proteoglycan, allowing its connection with signaling molecules in the cytoplasm [18].

Particularly, this domain of CSPG4 is characterized by two threonine residues which represent different phosphoacceptor sites which, based on the differential phosphorylation, drive different signaling cascades associated with different cellular events [51]. CSPG4 does not have intrinsic catalytic activity, therefore it actively participates in signal transduction by acting as a co-receptor for sustaining the activation of downstream signaling pathways such as FAK and MAPK pathways [52].

Moreover, by directly binding to ECM components, such as fibronectin and collagen, it is involved in enhanced cell adhesion and invasiveness [14,53]. Finally, CSPG4 mediates the activation of the α3β1 integrin/PI3K signaling and its downstream targets, promoting cell chemoresistance and survival [54].

CSPG4 was first discovered in 1981 [55] to be overexpressed on malignant melanoma cells. More recently, interest in characterizing its role in other tumor entities has been growing, due to its multifaceted functions and its significant contribution to tumor malignancy. Among different aggressive tumor histotypes for which no targetable antigens have been identified so far, OSA represents a major challenge. Our recent findings strongly support the rationale for exploiting CSPG4-targeted immunotherapies in the treatment of OSA. A deeper understanding of the mechanisms of action, along with the identification of predictors of therapeutic response or resistance, would enable the rational design of combination (immune)therapies. Such strategies could target OSA on multiple fronts, ultimately aiming for durable protection and complete tumor control.

## 4. xCT (SLC7A11)

Another candidate target that plays a multifaceted role is xCT, as it is involved in acquisition/maintenance of most of the “hallmarks of cancer” and is overexpressed in various cancer types, including OSA, where its expression inversely correlates with overall survival [20,56]. Encoded by the SLC7A11 gene, it is a 12-pass transmembrane protein that forms the antiporter system xc- together with its binding partner 4F2 cell-surface antigen heavy chain (4F2hc, SLC3A2). System xc- exchanges intracellular glutamate with extracellular cystine. Exported glutamate potentiates oncogenic signaling by acting on selective receptors expressed by cancer cells [57], while, inside of the cell, imported cystine is converted to cysteine to synthetize GSH, thereby detoxifying reactive oxygen species (ROS) to help tumor cells maintaining the redox balance and resist oxidative stress, including forms of cell death like ferroptosis [20]. Beyond its metabolic role, xCT has been implicated in immune evasion and extracellular vesicle (EV) dynamics [58]. xCT is thus an important determinant of tumor progression and radio/chemo-resistance, essential functions of cancer stem cells (CSCs) [59,60,61]. Indeed, xCT expression is increased in CSCs [62], where it physically interacts with CSC markers such as CD44, MUC1, IL1RAP [63,64,65,66]. A recent study found that a transcription factor, MLX, implicated in the regulation of stem cell functions in other contexts [67], positively regulates xCT expression in OSA via a super-enhancer-driven mechanism and predicts a poor prognosis [20]. This and other recent studies [20,21,22,23,24,25] have highlighted the feasibility and clinical promise of inhibiting xCT expression in OSA, thereby inducing ferroptosis, a form of regulated cell death driven by iron-dependent lipid peroxidation [33]. Several compounds have been identified that exploit this vulnerability in OSA cells: Sulforaphane promotes the interaction between p62 and xCT, leading to the autolysosomal degradation of xCT [21]; Baicalin and Shikonin induce NRF2 ubiquitin degradation, which suppresses downstream targets expression, including xCT [22,23]; Sulfasalazine (SAS), an FDA- and EMA-approved drug, inhibits xCT antiporter function [24].

Additionally, we have developed several vaccines able to target xCT and to prevent the development of metastases in preclinical models of breast cancer [62,68,69,70], thus providing a valuable therapeutic strategy to target xCT and develop combined therapies [71,72], potentially applicable to OSA.

## 5. TLR2

TLR2 is a member of the Toll-like receptor family, a group of PRRs that detect pathogen-associated molecular patterns (PAMPs), i.e., common molecular signatures of pathogens, and damage-associated molecular patterns (DAMPs) released upon tissue stress or damage. The triggering of TLRs leads to the activation of multiple signaling pathways, including the MAPK, NF-kB and interferon regulatory factors (IRFs) pathways, which result in the production of inflammatory cytokines and/or type I interferons (IFNs) [73].

TLRs are traditionally known to be expressed by immune cell populations such as macrophages and dendritic cells. Their activation triggers the innate immune response, which in turn shapes the adaptive immune system, ultimately coordinating a system-wide defense against pathogens. However, TLRs can also be expressed by epithelial, endothelial and mesenchymal cells, influencing their proliferation and function [74,75,76]. Of note, several tumor cell types express TLRs, at levels comparable or higher than the corresponding normal tissue. In particular, TLR2 is expressed at high levels in several cancers, including gastric, pancreatic, breast, and prostate cancer, where its activation induces tumor progression and metastasis through different tumor cell-intrinsic mechanisms [29]. Interestingly, TLR2 is expressed on OSA cell lines [26], and we and others have demonstrated that TLR2 promotes cancer resistance to chemotherapy. Indeed, high TLR2 expression is associated with poor response to chemotherapy in pancreatic [77] and breast cancer [37] patients, suggesting that it could represent a good target to improve chemotherapy efficacy in OSA patients as well.

## 6. Investigating Hallmarks of OSA as Therapeutic Vulnerabilities for Novel Treatment Opportunities

**Figure 2:**
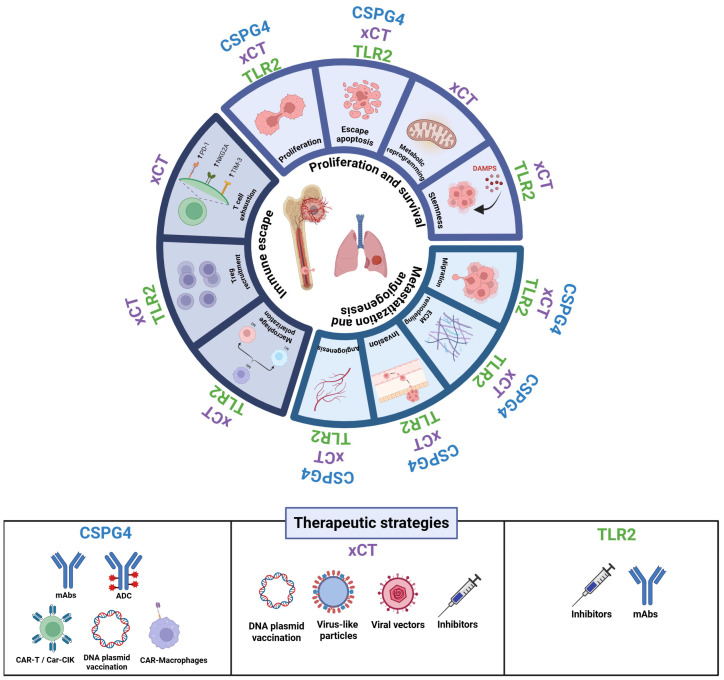
Roles of CSPG4, xCT, and TLR2 in tumor progression and associated therapeutic strategies. The figure illustrates the major biological processes influenced by CSPG4, xCT, and TLR2 in cancer progression, focusing on proliferation and survival, migration and angiogenesis, and immune escape. Each segment of the circular schema represents a cancer hallmark modulated by these molecules. The lower panel summarizes emerging therapeutic strategies targeting these molecules. Created in https://BioRender.com.

The extreme complexity of OSA makes it one of the few tumors for which no significant therapeutic advances have been achieved in the last 50 years [19,78,79]. OSA is highly heterogeneous at both the genomic and cellular level, and this heterogeneity often limits the efficacy of single-agent therapies, which rarely yield consistent benefits across patients [80,81,82,83]. Previous studies have broadened our understanding of OSA’s molecular landscape, revealing a high degree of genomic instability as a defining feature. Somatic copy-number variations, structural rearrangements, and diverse oncogenic pathway alterations contribute to this complexity. Nonetheless, the low tumor mutational burden, together with the scarcity of recurrent genetic alteration, makes the identification of effective and broadly applicable treatments particularly challenging. Based on these considerations, our aim is to revisit key hallmark features of OSA that may represent strategic therapeutic vulnerabilities, thereby paving the way for the development of novel treatment strategies. We will discuss how CSPG4, xCT, and TLR2 contribute to various tumor-supportive processes, and explore how these molecules could be exploited, either individually or in combination, to design novel precision treatment for OSA patients (Figure 2).

### 6.1. Sustaining Proliferation and Survival

Mutations in the TP53 gene—the most prevalent and strong predictors of OSA patients’ survival [84,85]—along with alterations in RB1 [86], contribute to defective DNA repair, dysregulated cell-cycle progression, and evasion of programmed cell death [87]. Together, these changes support a highly proliferative and apoptosis-resistant phenotype, aligning with one of the earliest hallmarks of cancer: sustained proliferative signaling and evasion of growth suppression.

While targeting mutated p53 and Rb1 in OSA is a scientifically sound idea, since their dysfunction helps drive tumor progression, clinical translation has lagged because of various obstacles, including tumor heterogeneity, and the complexity of downstream signaling networks, along with limited efficacy of p53-reactivating compounds and the absence of direct Rb1 restoration strategies [88,89,90,91].

Besides the defects in p53 and Rb1, diverse overexpressed oncogenes and growth-promoting receptors enable autonomous growth signaling in OSA, creating a permissive environment for malignant transformation and progression. Indeed, while normal cells tightly regulate growth in response to controlled external signals, transformed cells acquire the capacity of uncontrolled proliferation through different mechanisms which include the aberrant production of growth factor ligands and/or overexpression of their corresponding cell-surface receptors, resulting in a persistent activation of downstream pathways [92]. The development of molecular targeted therapies—among which tyrosine kinase inhibitors are the most promising—has been evolving in this direction to treat OSA patients [93,94].

Despite the molecular heterogeneity of OSA, some receptor tyrosine kinases—such as IGF-1R, EGFR, and PDGFR—are overexpressed or altered in a subset of patients (from 7% for IGF-1R to 90% for PDGFR). Targeted therapies against these receptors have been developed and tested; however, their clinical efficacy has so far remained limited [94,95,96].

In this context of uncontrolled cell growth, CSPG4 has recently emerged as an appealing target. We have demonstrated that CSPG4 supports the proliferative capacity of human OSA cells, as its transient downmodulation via small interfering (si)RNAs or monoclonal antibodies (mAbs) significantly impaired cell proliferation in vitro and enhanced the cytotoxic activity of DOXO [11,12]. These findings underscore the functional relevance of CSPG4 in sustaining tumor growth-associated signaling pathways in OSA. This observation is consistent with reports in other tumor types, where CSPG4 has been identified as a marker of aggressive, highly proliferative phenotypes, and its knock-down or mAbs-mediated blockade in established tumors results in significant growth inhibition [97,98]. In sarcoma models, Hsu et al. showed that tumor cells engineered to lack CSPG4 exhibited reduced proliferation and increased cell death [99]. Interestingly, while CSPG4 inhibition in established tumors decreased both tumor volume and proliferation rate, CSPG4 deletion at the time of tumor initiation paradoxically promoted the formation of larger tumors [99]. Transcriptomic analysis revealed that early CSPG4 deletion was associated with downregulation of insulin-like growth factor binding proteins (IGFBPs) and subsequent over-activation of IGF signaling, ultimately enhancing tumor growth [99]. To date, this remains the only evidence indicating that the impact of CSPG4 deletion may depend on the developmental stage of the tumor and the context of IGF signaling activation.

Mechanistically, the role of CSPG4 in regulating proliferation relies on its core protein—particularly the D2 extracellular domain—which interacts with growth factors, protects them from degradation, and facilitates receptor engagement, thereby sustaining signaling cascades such as the MAPK/ERK pathway. Although a direct correlation between CSPG4 and IGF-1R has not been demonstrated, associations with EGFR and PDGFR have been reported in other tumor types. In OSA, analysis of publicly available gene expression datasets suggests a potential association between CSPG4 and PDGFR expression (OS microarray dataset mixed osteosarcoma—Kuijjer-127-vst-ilmnhwg6v2 from the R2: Genomics Analysis and Visualization Platform, http://r2.amc.nl, accessed on 28 October 2025) [100,101]; while multiple factors likely influence their expression, the potential correlation may be biologically intriguing. Co-targeting CSPG4 together with its functional interaction partners may therefore enhance the inhibition of uncontrolled OSA proliferation, offering a promising avenue for the development of more effective therapeutic strategies.

By activating key survival signaling cascades—most notably the integrin/PI3K/AKT pathway [54]—CSPG4 has been shown to contribute to apoptosis resistance. CSPG4 may hence foster chemoresistance across multiple cancer types, including OSA, and its overexpression has indeed been implicated in impaired responses to several chemotherapeutic drugs in both human and canine OSA cell lines [11,12]. In our experience, targeting CSPG4 with a chimeric HuDo-CSPG4 vaccine after surgery and chemotherapy effectively delayed metastatic progression in vaccinated canine OSA patients as compared to controls treated with surgery and chemotherapy alone [11], suggesting that CSPG4 immune-targeting may overcome chemoresistance mechanisms ultimately improving outcomes.

To date, clinical trials evaluating this therapeutic approach in humans have not been performed. More broadly, conducting robust and adequately powered trials in OSA patients remains challenging due to the rarity of the disease, its rapid metastatic progression, and its biological heterogeneity. Therefore, results obtained in a highly translational OSA model such as dogs with spontaneously occurring tumors are particularly relevant [102]. Although we fully acknowledge the inherent species differences and recognize that findings from canine studies cannot be directly extrapolated to humans, numerous comparative oncology investigations have highlighted the genetic, histopathological, and clinical similarities between human and canine OSA [103,104,105], further supporting the reliability of this model. Moreover, exploring novel therapeutic strategies in the veterinary field remains an urgent and parallel need, given the severe outcomes associated with OSA in dogs [106].

Nevertheless, CSPG4 negative clones could anyway escape and give rise to disease evolution. In this context of drug resistance and evasion of cell death, xCT and TLR2 also could provide a relevant contribution and considering their (over)expression in OSA, there could be a strong rationale for combinatorial approaches.

Notably, these three molecules do not act in isolation. CSPG4-mediated integrin/PI3K/AKT activation [54] intersects directly with TLR2-driven PI3K/AKT [107,108] and NF-κB signaling [109], providing multiple, partially redundant pro-survival inputs that can reinforce one another under cytotoxic stress. In parallel, xCT maintains the redox balance required for sustained PI3K/AKT activity and prevents ROS-induced interruption of these pathways. Together, these mechanisms suggest that CSPG4, xCT, and TLR2 may cooperate to generate convergent, synergistic survival signals that enable OSA cells to resist apoptosis and therapy.

Moreover, TLR2-driven NF-κB activation [37] further reinforces survival by upregulating xCT [38], thereby coupling inflammatory signaling to GSH-dependent redox balance and ferroptosis resistance, which collectively support OSA cell persistence under stress and therapy.

xCT contributes to cancer cell intrinsic resistance to cell death by maintaining redox homeostasis and preventing both oxidative stress–induced apoptosis and ferroptosis [110]. In addition, xCT activity supports NADPH regeneration and stabilizes redox-sensitive survival pathways, including Bcl-2 and NF-κB signaling, while mitigating endoplasmic reticulum stress-associated apoptosis [38,111,112,113]. Through the export of glutamate, xCT also influences amino acid sensing and mTORC1 activation, enabling metabolic adaptation and survival under nutrient-limiting conditions [114,115]. Beyond these intrinsic mechanisms, xCT plays a pivotal role in protecting tumor cells from therapy-induced oxidative damage [116]. Numerous studies have shown that xCT overexpression confers resistance to a wide range of chemotherapeutic agents, including CDDP and DOXO [117,118,119,120,121,122]. Conversely, inhibition of xCT, whether genetically or pharmacologically, restores sensitivity to these treatments, while supplementation with GSH or its precursors diminishes therapeutic efficacy [20,24]. Large-scale pharmacogenomic analyses have indicated that the correlation between xCT expression and drug resistance can be as predictive of drug response as classical multidrug resistance genes such as ABCB1 and ABCC1 [123,124,125].

In the development of resistance to chemotherapeutic agents, particularly DOXO, widely used in multi-chemotherapeutic approaches for OSA patients, TLR2 could play a pivotal role. Following treatment with immunogenic cell death-inducing drugs such as DOXO, cancer cells release HMGB1, a DAMP that serves as a ligand for TLR2. Binding of HMGB1 to TLR2 triggers multiple signaling cascades, including the NF-κB pathway, which promotes CSC self-renewal and chemoresistance. Consequently, genetic or pharmacologic inhibition of TLR2 significantly reduces CSC frequency and enhances the efficacy of chemotherapy [36,37]. HMGB1 released from dying tumor cells can also activate TLR2 on neighboring viable cells, stimulating YAP/HIF-1α signaling and promoting dedifferentiation towards a stem-like phenotype, a process that underlies tumor relapse and resistance to chemo-radiotherapy [126]. This TLR2-dependent pathway has been implicated in resistance mechanisms across several tumor types, including OSA [127], where therapy-induced DAMP release and TLR2 activation contribute to autophagy, pro-survival transcriptional programs, and maintenance of stemness [128]. Consistent with this, inhibition or genetic deletion of TLR2 enhances tumor sensitivity to chemotherapy in preclinical models, underscoring its causal role in therapy resistance and highlighting TLR2 as a promising target [73]. Further supporting this link in OSA, the TLR2’s downstream adaptor MyD88 is overexpressed in OSA cells compared with normal bone tissue. Importantly, patients with MyD88-positive OSA exhibit significantly lower 5-year survival rates than MyD88-negative counterparts, and inhibition of MyD88 impairs OSA cell proliferation while promoting apoptosis [129]. These findings collectively confirm that the HMGB1–TLR2–MyD88 axis contributes to OSA cell survival and resistance to conventional therapies.

### 6.2. Metastasization and Angiogenesis

The ability to give rise to metastasis is a key feature defining malignancy and is responsible for the vast majority of cancer-related deaths worldwide. The metastatic process comprises a series of coordinated events collectively known as the “invasion-metastasis cascade”.

The most common site of cancer cell dissemination in OSA is the lung, accounting for approximately 80% of metastatic cases and significantly contributing to treatment failure and mortality. The pulmonary metastasis process can be divided into three main stages, including escape of cancer cells from primary tumor in the bone, transit within circulation system, and colonization and establishment of disseminated cells in the lung. As a first step, OSA cells must acquire a migratory and invasive phenotype—a hallmark of cancer specifically associated with tumor progression and metastasis. This enables cells to migrate away from the primary tumor site and infiltrate surrounding tissues. Recent in vitro and in vivo findings by our group [11] support a functional role of CSPG4 in promoting this phenotype in OSA cells. Specifically, transient downregulation of CSPG4 expression or immune targeting through mAbs led to a significant reduction in cell migration in vitro. Moreover, CSPG4-vaccine-induced immunity exerts a protective effect able to inhibit metastatic dissemination of OSA cells to the lungs in a human OSA xenograft model [11]. These findings underscore the potential role of CSPG4 in regulating the metastatic phenotype in OSA. Consistently, anti-CSPG4 vaccination in dogs has been shown to slow metastatic progression, and one possible mechanism underlying this effect may involve vaccine-induced antibodies that interfere with CSPG4-mediated cell migration and invasion, thereby limiting the metastatic potential of tumor cells [11].

From a mechanistic point of view, the process of cancer invasion and migration is critically dependent on the destruction and degradation of basement membrane and ECM, which are catalyzed by extracellular proteases, mainly through the upregulation of different members of the matrix metalloprotease (MMP) family. There is substantial evidence that MMP-2 and MMP-9 are among the most involved MMPs in mediating this process in OSA [130,131,132,133].

Thanks to its structure, CSPG4 has a strong ability to interact with many ECM components, influence their organization, and modulate signaling. Multiple studies in aggressive tumor types beyond OSA provide strong evidence that CSPG4 can participate in MMP activation through direct binding via its CS chains, thereby promoting local cancer cell invasion. For example, in melanoma, the binding of pro-MMP-2 (the inactive precursor) to CSPG4 facilitates its localization at the cell membrane near other MMP-activating components that convert it into active MMP-2, leading to localized ECM degradation at the invasive front. The direct connection between CSPG4 and MMP-2 activation documented in different tumor types, suggests that a similar mechanism may operate in OSA, although experimental confirmation in this tumor context is still lacking, albeit intriguing. The positive correlation between CSPG4 overexpression and MMP-9 in OSA is also supported by public available datasets (OS microarray dataset mixed osteosarcoma-Kuijjer-127-vst-ilmnhwg6v2 from the R2: Genomics Analysis and Visualization Platform, http://r2.amc.nl, accessed on 28 October 2025) [100,101], suggesting a potential biological link raising the possibility that CSPG4 contribute to enhanced ECM remodeling and invasive potential in OSA cells, warranting further investigation.

In addition, CSPG4-dependent integrin/FAK/Src signaling may functionally intersect with TLR2-mediated NF-κB and PI3K/AKT activation, both of which promote cytoskeletal reorganization and MMP expression [52,107,108,109]. xCT-dependent redox control further supports these processes by sustaining ROS-sensitive components of the invasion machinery. These converging pathways may collectively enhance the invasive and metastatic potential of OSA cells.

High xCT expression correlates with increased invasion, dissemination, and poor prognosis across multiple tumor types, including OSA [20,56]. Mechanistically, xCT inhibition—genetic or pharmacologic—reduces migration and invasion by redistributing caveolin-1 and β-catenin, activating p38 MAPK, and elevating ROS levels, thereby impairing ECM interactions and metastatic potential [59,134]. Super-enhancer-driven transcription factors such as MLX activate SLC7A11 transcription, while the histone demethylase KDM4A epigenetically elevates it, inhibiting ferroptosis and enhancing migration and lung colonization [20,135]. Additionally, the circular RNA circKIF4A sponges miR-515-5p to prevent SLC7A11 suppression, thereby supporting oxidative stress resistance and metastatic dissemination [136]. Beyond tumor-intrinsic functions, xCT in the TME contributes to metastasis by fostering immune evasion (see below). Collectively, these findings indicate that xCT integrates redox regulation, oncogenic pathways, and immune modulation to support metastatic progression.

Similarly, TLR2 expression in tumor cells has been associated with tumor progression as well as enhanced invasive and metastatic potential across different cancer types [29]. Importantly, it has been recently reported that osteoblasts and OSA cells express TLR2, along with related receptors such as TLR4 and RAGE, and that their activation by HMGB1 promotes cell migration [137]. HMGB1, which can be released upon tissue damage and cell death, has been shown by Ming-Jing Li et al. to trigger a signaling cascade by binding to its receptors, acting as a regenerative cytokine and promoting osteoblasts migration by TLR2/4-dependent pathways [137]. Mechanistically, TLR2 activation in tumor cells can promote migration and invasion through multiple converging pathways. For instance, TLR2 up-regulation drives the MyD88-dependent activation of NF-κB, which increases expression of pro-inflammatory cytokines and matrix-degrading enzymes (e.g., MMP-2 and MMP-9) and activates the epithelial-mesenchymal transition (EMT) program, thereby enhancing motility and invasive potential [109]. TLR2 also engages the PI3K/AKT cascade, contributing to survival, EMT, cytoskeletal re-organization and enhanced motility [107,108]. Moreover, TLR2’s influence is reinforced via downstream MAPK/ERK pathways and integrin/FAK/Src-mediated adhesion dynamics, thereby facilitating detachment, migration and ECM invasion [138]. These mechanistic links delineate how TLR2 on tumor cells (or within the TME) may act as a pro-metastatic switch by coordinating inflammation, survival and motility pathways. Additionally, by inducing NF-κB-dependent xCT expression, TLR2 links innate inflammatory activation to metabolic immune escape, since enhanced GSH synthesis and ferroptosis resistance enable tumor cells to withstand ROS-mediated cytotoxicity from immune effector cells.

An essential supporting process for metastasis is angiogenesis, another well-recognized hallmark of cancer. Angiogenesis is a crucial feature of OSA tumorigenesis, a complex and dynamic process regulated by the balance of angiogenic and anti-angiogenic factors under physiological conditions. In the TME, this homeostasis is disrupted, resulting in excessive angiogenesis that provides nutritional support to the tumor, thereby promoting growth, invasion, and metastasis.

In OSA, the rapid tumor growth often results in chronic hypoxia within the TME, which is closely associated with enhanced EMT and tumor stemness, features linked to a more aggressive and metastatic phenotype. Given their shared influence on hypoxia-responsive, redox-regulated, and PI3K/AKT-dependent programs, CSPG4, xCT, and TLR2 may cooperate to amplify angiogenic signaling within the OSA TME, thereby supporting vascular remodeling and metastatic dissemination.

Hypoxia stabilizes the transcription factor HIF-1α, which in turn induces the expression of angiogenic factors, primarily vascular endothelial growth factor (VEGF). VEGF rapidly stimulates its receptor (VEGFR) on OSA and endothelial cells, promoting neo angiogenesis that supplies nutrients to the tumor. While VEGF is the “master regulator” of new blood vessel formation, PDGF secreted by endothelial and tumor cells is responsible for vessel maturation through the recruitment of pericytes. Proper PDGF signaling via PDGFR is crucial for the stabilization and maturation of new vessels. In this setting, CSPG4 could have a prominent supportive role. On one hand, CSPG4 could serve as co-receptor sustaining the activation of angiogenic pathways by modulating angiocrine factors as fibroblast growth factor (FGF)-2 and PDGF once bound to CSPG4 [51]. On the other hand, CSPG4 may directly control the behavior of the neovasculature [53]. In different tumor settings, CSPG4 has been shown to be upregulated at both mRNA and protein levels under chronic hypoxic conditions in venular pericytes, sustaining their activation during hypoxia-induced angiogenesis and vascular remodeling in growing tumors [44]. CSPG4 overexpression enhances pericyte recruitment and attachment to endothelial cells, while its low expression on mature vasculature supports its crucial role in early angiogenesis and vascularization [51]. Stable CSPG4 knock-down results in less vascularized tumors in glioblastoma [97] and uveal melanoma models [139]. Although data specifically addressing CSPG4 role in OSA remain limited, available evidence suggests that CSPG4-expressing pericytes are important in maintaining bone sarcoma vasculature and stabilizing blood vessels, thereby influencing tumor growth and progression [140]. Considering the extensive vascularization that characterizes advanced OSA, it is worth investigating the potential role of CSPG4 in this tumor to develop novel targeted therapies aimed at impairing neovascularization and ultimately hampering OSA progression.

Anti-angiogenic drugs have already been tested in OSA, both at preclinical and clinical settings. The anti-VEGF mAb Bevacizumab has shown strong activity against the primary tumor but not against lung metastasis development in a mouse model of human OSA [141]. However, Bevacizumab, either alone or combined with multi-drug chemotherapy, failed to provide a survival benefit in a Phase II clinical trial [142]. Volz and colleagues found that a CSPG4 polymorphism may predict shorter progression-free survival in colorectal cancer patients treated with Bevacizumab in combination with chemotherapy [143], opening new possibilities for designing improved therapeutic interventions for OSA patients.

Specific studies on OSA support a tumor-promoting vascular role for xCT, as its expression is maintained by super-enhancer-driven MLX, and its inhibition sensitizes tumors to ferroptosis while attenuating pro-angiogenic signaling [20,33]. Several lines of evidence implicate xCT not only in sustaining pro-angiogenic programs but also in shaping vascular architecture to enhance tumor access to the circulation, with direct evidence that its inhibition reduces tumor vascular density. From a mechanistic point of view, extracellular glutamate exported by xCT acts in a paracrine manner on stromal and endothelial compartments, where glutamate receptor activation and redox-sensitive HIF-1α stabilization promote VEGF expression and endothelial sprouting [122,144,145]. xCT expression is sustained by upstream regulators such as ATF4 and ERO1α, integrating stress and ER-folding pathways into pro-angiogenic programs. In vivo, suppression of ERO1α reduced VEGF production and CD31^+^ vessel density in xenografts, whereas xCT re-expression restored vascularization [146].

TLR2 may act as an important modulator of tumor angiogenesis through both direct and indirect mechanisms. Indeed, TLR2 is expressed not only by innate immune cells but also by non-hematopoietic compartments, including endothelial cells. Upon activation by DAMPs, endothelial TLR2 can promote pro-angiogenic behavior. For instance, McCoy et al. reported that TLR2 activation in endothelial cells increases the recruitment of pro-angiogenic immune cells and facilitates new vessel formation within the TME in a prostate cancer model [75]. Another study reported the activation of TLR2 in endothelial cells by DAMPs produced during inflammation and wound healing but also in highly vascularized melanomas. These end products of lipid oxidation, v-(2-carboxyethyl) pyrrole (CEP) and other related pyrroles, have been shown to drive an angiogenic response independently of VEGF [147]. Importantly, CEP and related oxidative derivatives can arise because of lipid peroxidation triggered by elevated ROS and glutamate export in xCT-overexpressing tumor cells, thereby establishing a mechanistic bridge between xCT activity, oxidative stress, and TLR2-mediated angiogenic signaling. In addition, we have previously demonstrated that DAMPs released following chemotherapy in breast cancer can activate TLR2 expressed by tumor cells, leading to NF-κB activation and the secretion of pro-angiogenic factors, including VEGF [37]. These findings highlight a dual mechanism of action for TLR2, mediating VEGF release from tumor cells and acting independently of VEGF in endothelial cells to promote vascularization.

Together CSPG4, xCT and TLR2 appear to play significant roles in the angiogenic process, which is critically important in OSA for supporting cancer cell dissemination as well as metastasis formation. Since lung metastases are the leading cause of mortality in OSA patients, these findings provide a strong rationale for further investigating these molecules and the consequences of their targeting as potential innovative strategies to prevent metastasis.

### 6.3. Immune Evasion

Immune evasion represents a fundamental hallmark of cancer, enabling tumor cells to escape recognition and destruction by the host immune system through a range of mechanisms—either intrinsic (for example, expression of immune-checkpoint ligands) or extrinsic (for example, recruitment of immunosuppressive cells into the TME). Advances in immunotherapy have led to novel strategies to harness the host’s anti-tumor immune response, including immune checkpoint inhibitors (ICIs), engineered T cells and anti-tumor vaccines, which have shown promising results in a variety of solid tumors [148]. However, in the context of OSA, these approaches have generally failed to produce meaningful clinical benefit. This lack of efficacy appears to result from a combination of tumor-intrinsic properties—such as pronounced heterogeneity and low mutational burden—and tumor-extrinsic factors, including a consistently immunologically “cold,” suppressive microenvironment. Therefore, novel and more precise treatments are currently under intense investigation in this setting.

Importantly, several immune-modulatory activities of CSPG4, xCT, and TLR2 converge on overlapping inflammatory pathways such as NF-κB and HIF-1α, suggesting that these molecules may collectively reinforce immunosuppressive network loops within the OSA microenvironment.

It is increasingly evident that OSA derives limited benefit from classical ICI therapies. For instance, blockade of the PD-1/PD-L1 axis in localized OSA has shown minimal activity in Phase II clinical trials [149]. The main limitations likely include heterogeneous and often low expression of PD-L1, poor T cell infiltration, and the presence of multiple redundant immune escape pathways. Interestingly, in a melanoma model—where a similarly variable PD-L1 expression pattern is typically observed—a bispecific antibody targeting both PD-L1 and CSPG4 displayed enhanced, selective binding to tumor cells co-expressing these molecules, accompanied by increased T-cell activation [150]. This finding raises the hypothesis that CSPG4 may serve as a co-target to improve T-cell-mediated anti-tumor responses in tumors with heterogeneous PD-L1 expression. CSPG4 may contribute to immune evasion, potentially also in other tumor types, including OSA. Supporting this notion, CSPG4 overexpression has been associated to reduced immunogenicity and cytotoxic T-cell responses in a soft tissue sarcoma model [151].

Another immune checkpoint ligand, B7-H3 (CD276), is highly expressed in OSA, often exceeding PD-L1 levels, and its expression correlates with poor outcome and reduced CD8^+^ T-cell infiltration [152,153,154,155]. We recently demonstrated in an OSA xenograft model that adoptively transferred CSPG4-vaccine-induced T cells effectively infiltrated and delayed or regressed tumors in a subset of cases, whereas non-responding tumors selectively up-regulated B7-H3 (but not PD-L1), thereby implicating B7-H3 in immune escape in this setting [11]. These findings provide a strong rationale for therapeutic strategies combining CSPG4-directed vaccination with B7-H3 blockade, to simultaneously promote antigen-specific T-cell activation and alleviate checkpoint-mediated suppression.

Given the multiplicity and redundancy of immune escape mechanisms in OSA, additional targets may further enhance the efficacy of immune-based approaches. Molecules such as TLR2, which mediates crosstalk between tumor cells and the immunosuppressive TME and influences the polarization of immune cells toward pro- or anti-tumor phenotypes [29], represent particularly attractive candidates for inclusion in combinatorial regimens aimed at achieving durable tumor control.

Although studies directly addressing TLR2 in OSA are scarce, evidence from other TLR family members highlights the therapeutic potential of modulating TLR signaling to reprogram the suppressive TME. For example, TLR9 is highly expressed in most OSA specimens and correlates with tumor progression [26]. Despite its constitutive expression, intratumor activation of TLR9 with CpG ODN 2395 or SD101 potently inhibited primary tumor growth and triggered systemic abscopal effects, prolonging survival in murine OSA models. This benefit was immune-mediated, driven by the repolarization of M2-like macrophages towards an anti-tumor phenotype and the systemic expansion of activated cytotoxic CD8^+^ T cells [156]. Similarly, activation of TLR4 with a detoxified agonist (Lipo-MP-LPS) suppressed OSA growth and lung metastasis in a CD8^+^ T cell-dependent manner, accompanied by M1 macrophage enrichment in primary and metastatic sites [157]. These underscore the dual nature of TLR signaling in cancer, capable of either sustaining tumor-promoting inflammation or stimulating robust anti-tumor immunity, depending on the receptor subtype, cell context and timing of activation.

Among TLRs, TLR2 stands out for its frequent association with immunosuppressive reprogramming. It is expressed on multiple regulatory and myeloid cell populations, including T regulatory cells (Tregs), myeloid derived suppressor cells (MDSCs), macrophages, and neutrophils. Activation of TLR2 on these cells fosters the establishment of an immunosuppressive milieu that can facilitate primary tumor growth and metastatic colonization [29].

In breast cancer mouse models, TLR2 stimulation in Tregs promotes their expansion and accelerates tumor progression, while TLR2 activation in macrophages triggers cytokine secretion that further recruits Tregs to the TME [158,159]. Within this context, Tregs mediate suppression through cytokines such as IL-10, which inhibits CD8^+^ T-cell activity [29]. Similarly, TLR2 engagement in B lymphocytes drives their differentiation into regulatory B cells (Bregs), leading to IL-10 production and suppression of T cell responses [160]. In CD4^+^ T cells, endogenous ligands such as heat shock protein (HSP)90 or other DAMPs released from tumor cells can activate TLR2, triggering an IL-6-dependent cascade that enhances IL-10 and IL-21 secretion, thereby reinforcing immunosuppression [161]. Moreover, TLR2 signaling in bone marrow progenitors promotes their differentiation into MDSCs, which accumulate in the TME, secrete pro-tumoral cytokines, polarize macrophage toward an M2 phenotype, and suppress effector T-cell response. Finally, DAMPs such as HMGB1 and serum amyloid A1, whose high levels in serum are associated with poor prognosis in OSA and breast cancer patients, can activate TLR2 on neutrophils, leading to additional immunosuppressive effects [162,163,164].

Together, these findings delineate a cohesive picture in which TLR2 acts as a key mediator of tumor-induced immunosuppression across several cancers. Given that conventional immunotherapies have achieved limited success in OSA patients, targeting TLR2 offers a strategy to remodel the immune landscape and improve therapeutic efficacy. Indeed, TLR2 inhibition synergizes with chemotherapy in pre-clinical breast cancer models, reinstating an immune permissive TME and enhancing treatment response [29]. These results suggest that combining TLR2 blockade with chemotherapy or immunotherapy could represent a promising approach to overcoming immune evasion and improving outcomes in OSA.

The OSA immunosuppressive TME is further shaped by metabolic reprogramming, in which xCT plays a pivotal role. OSA cells exhibit strong dependence on glucose and glutamine for proliferation and survival, and they can flexibly switch between these substrates under nutrient-limiting conditions [165,166,167,168]. xCT supports this metabolic plasticity by exporting glutamate, thereby linking glutamine metabolism to redox homeostasis and facilitating adaptation to oxidative and nutrient stress [169,170]. However, excessive xCT activity can heighten cellular reliance on glucose and sensitize tumor cells to glucose deprivation, leading to impaired mitochondrial function and disulfide-induced cell death (disulfidptosis) [171]. Thus, xCT enhances tumor metabolic fitness while simultaneously creating potential vulnerabilities.

Beyond its metabolic functions, xCT serves as a critical immunometabolic node. Although direct studies in OSA are limited, evidence from other tumor models strongly indicates that xCT modulates the TME. In triple-negative breast cancer (TNBC), xCT depletion in tumor cells increases intratumor CD8^+^ T-cell infiltration and reduces circulating MDSCs, impairing metastatic colonization [134]. By analogy, xCT overexpression in OSA could limit effector T-cell activity through converging metabolic and signaling mechanisms.

First, excessive export of glutamate by tumor cells promotes the formation of an “immunosuppressive synapse” between tumor cells and infiltrating T cells. Elevated extracellular glutamate leads to heightened stimulation metabotropic glutamate receptors—particularly mGluR4—on T cells, reducing intracellular cAMP levels and attenuating the PKA–CREB pathway, which collectively suppress T-cell activation, proliferation, and cytokine production [172]. T cells also express other metabotropic (mGluR1, mGluR5) and ionotropic glutamate receptors that modulate activation; however, glutamate overload can disrupt Ca^2+^ fluxes, destabilize lipid rafts, and impair CD3ζ and ZAP70 phosphorylation, thereby weakening TCR signalosome assembly and downstream activation programs [173]. These effects highlight xCT-driven glutamate release as a direct mechanism for dampening TCR signaling.

Second, high xCT activity decreases extracellular cystine availability. This directly competes with T cells and macrophages, which import cystine and convert it into extracellular cysteine needed to sustain T-cell activation. In a cysteine-poor microenvironment —further exacerbated by MDSCs that import cystine but fail to release cysteine [174]—CD8^+^ T cells experience inadequate cysteine supply, reduced GSH synthesis, elevated oxidative stress, and increased susceptibility to exhaustion or ferroptotic cell death [175]. These combined metabolic constraints reinforce an immunosuppressive niche.

xCT may also influence regulatory immune populations. In other tumor types, xCT-mediated glutamate release promotes Tregs expansion via mGluR1 signaling and stabilizes HIF-1α, which drives PD-L1 expression and chemokine-mediated recruitment of MDSCs and macrophages, polarizing the latter to an M2-like phenotype [176,177]. While not directly demonstrated in OSA, these mechanisms provide a plausible link between xCT activity and immune evasion in this tumor. Finally, suppression of xCT in cancer cells sensitizes them to ferroptotic death, releasing DAMPs that can activate dendritic cells (DCs) and CD8^+^ T cells, thus potentially enhancing anti-tumor immunity. In preclinical models, xCT inhibition synergizes with checkpoint blockade or chemotherapy, suggesting a therapeutic window in which xCT-targeted interventions could reinforce immune-mediated tumor control [62,178,179].

Taken together, the available OSA-specific data, combined with mechanistic insights from other tumors, position xCT as a key metabolic–immune hub in OSA. Its targeting may not only impair tumor cell survival but also modulate the TME to favor effector T-cell function, making it a promising complement to CSPG4- and/or TLR2-directed immunotherapeutic strategies. More broadly, the collective evidence indicates that CSPG4, xCT, and TLR2 contribute to immune evasion through partially overlapping and mutually reinforcing inflammatory and metabolic pathways. Their combined targeting may therefore disrupt interconnected circuits within the OSA microenvironment that sustain tumor progression.

## 7. Therapeutic Targeting and Future Directions

In this review, we have proposed three molecules—CSPG4, xCT, and TLR2—as novel and mechanistically complementary targets for OSA. Each represents a distinct facet of OSA pathophysiology: CSPG4 as a surface antigen primarily associated with tumor cell-intrinsic malignant properties, xCT as a central metabolic and immunometabolic node sustaining redox balance, metabolic fitness, and immune suppression, and TLR2 as a dual regulator that supports tumor cell proliferation and therapeutic resistance while also shaping the TME.

Despite major progress in immuno-oncology, many tumors, including OSA, remain poorly immunogenic due to their low mutational burden and highly immunosuppressive microenvironment. Consequently, identifying tumor-associated antigens (TAAs) that are functionally relevant to OSA progression and shared across patients is essential for developing effective immunotherapies. The strategies discussed herein—ranging from antibody- and vaccine-based approaches to metabolic and innate immune modulation—illustrate how multi-target interventions may overcome the intrinsic resistance of OSA to immune attack and pave the way for more durable clinical responses.

We have recently identified CSPG4 as a promising immunotherapeutic target for OSA management [11,12]. Over the last decades, several immunotherapeutic approaches targeting CSPG4 have been developed and tested in different tumor types [50,180,181,182], with melanoma being the most extensively studied. Early investigations demonstrated the anti-tumor effects of anti-CSPG4 mAbs in vitro and in vivo [18,183,184]. De Bruyn and coworkers showed that an anti-CSPG4:TRAIL fusion protein, generated by linking tumor necrosis factor related apoptosis-inducing ligand (TRAIL) to an anti-CSPG4 scFv derived from mAb 9.2.27, exhibited significant antitumor activity [185]. Moreover, combinatorial approaches with standard-of-care therapies have already been explored—such as anti-CSPG4 mAbs combined with the BRAF inhibitor PLX4032 in melanoma—showing synergistic impairment of malignant cell properties and supporting the rationale for combinatorial administration in vivo [186].

Recently, CSPG4-specific chimeric antigen receptors (CARs) have been developed and tested preclinically, demonstrating striking efficacy in controlling tumor growth in human xenografts [187,188]. However, self-TAAs overexpressed on tumor cells, such as CSPG4, are generally well tolerated by the host immune system. To overcome this, CSPG4 targeting has been pursued through antigen mimicry, leveraging anti-idiotypic antibodies. For instance, the anti-idiotypic mAb MK2-23, which mimic the epitope recognized by anti-CSPG4 mAb 763.74, induced specific antibody responses in metastatic melanoma patients that correlated with prolonged survival and reduced metastatic spread [189].

One major limitation of these strategies is their poor induction of robust cellular immunity, which is essential for controlling established tumors. In this context, we tested, for the first time in OSA, a DNA plasmid-based immunization strategy using a chimeric HuDo-CSPG4 vaccine designed to elicit both humoral and cellular immune responses against CSPG4. This vaccine induced potent CD8^+^ T-cell and antibody responses that delayed tumor progression and metastasis upon adoptive transfer in immune-deficient mice bearing human OSA xenografts. Moreover, the HuDo-CSPG4 vaccination broke immune tolerance toward the self-canine CSPG4 antigen, inducing functionally effective immunity in dogs with spontaneous OSA [11]. In our pilot veterinary study, we also demonstrated the feasibility of this therapeutic platform and its potential to be effectively integrated with standard treatment modalities. In the OSA setting, where surgery and chemotherapy remain the gold standard, the promising evaluation of anti-CSPG4 DNA vaccination in an adjuvant context following standard treatments [11] underscores the value of combining innovative approaches with established clinical protocols. Such combinatorial strategies are important not only for enhancing the efficacy of individual therapies but also for enabling the incorporation of novel interventions into existing treatment frameworks. This integrative perspective supports the goal of providing patients with the most effective therapeutic options while reinforcing standard care through scientifically grounded innovation.

The demonstration of safety, immunogenicity, and clinical benefit of HuDo-CSPG4 vaccination in canine OSA patients represents a significant step forward consistent with the principles of comparative oncology, which aims to accelerate translational progress from preclinical studies to human clinical settings [190]. Comparative oncology continues to gain recognition for OSA research, as the disease’s rarity and young patient population limit extensive clinical testing of novel drugs. The close similarity between human and canine OSA [103,104,105] further strengthens the translational value of comparative oncology approaches. In general, DNA vaccination is a versatile platform and may be effectively adapted to target various tumor antigens [191]. Nonetheless, this first proof-of-concept study demonstrating immune-targeting of CSPG4 in OSA warrants further investigation. Future efforts should focus on identifying predictive biomarkers of vaccine response and improving vaccine design to enhance immunogenicity, for instance by developing multi-epitope DNA or mRNA vaccines. However, monotherapy approaches remain vulnerable to tumor immune escape, which can result from antigen loss or intratumoral heterogeneity driven by clonal evolution [192]. Therefore, incorporating additional targets, especially for complex, treatment-resistant malignancies such as OSA, is likely required. Rational multi-targeting strategies that simultaneously act on tumor cells and the TME are critical to achieve durable responses and align with the principles of precision medicine, tailoring interventions to the molecular and immunological profiles of individual tumors.

In this framework, modulation of tumor metabolic dependencies has emerged as a promising complementary strategy. Pharmacological inhibition of xCT has been extensively explored using small-molecule inhibitors such as SAS, erastin, and sorafenib, all of which reduce cystine uptake and disrupt intracellular redox homeostasis, sensitizing tumor cells to oxidative stress and ferroptotic cell death in preclinical models [117,193,194]. SAS, an FDA-approved anti-inflammatory drug, was one of the first agents identified to inhibit xCT, leading to GSH depletion and ROS accumulation. However, its limited potency, specificity, and pharmacokinetic profile have hindered its translation to oncology [27]. Conversely, erastin and sorafenib, originally described as RAS-selective lethal compounds [195] and a multi-kinase inhibitor [196], respectively, act as potent ferroptosis inducers by directly or indirectly suppressing xCT function, resulting in lethal lipid peroxidation [197]. Despite compelling preclinical evidence, clinical implementation of these drugs as xCT inhibitors remains constrained by systemic toxicity and redox imbalance in normal tissues due to off-target effects.

These toxicity concerns become even more relevant in the context of multi-targeting strategies. Systemic xCT inhibition can impair antioxidant defenses in normal organs—particularly the liver and pancreas—raising the risk of overlapping toxicities when combined with other targeted or immune-modulating agents. Similarly, TLR2 is broadly involved in normal immune regulation, where systemic modulation could lead to unwanted inflammation or immune-related adverse effects. As a result, the therapeutic window may narrow substantially in combinatorial regimens. To address these limitations, tumor-selective delivery systems are increasingly being investigated. Antibody-drug conjugates (ADCs), ligand-directed vehicles, and engineered nanoparticles can restrict the biodistribution of xCT-directed therapeutics to tumor tissues, thereby minimizing systemic exposure. In OSA, the overexpression of CSPG4 represents an attractive targeting moiety for such delivery platforms. CSPG4-directed nanoparticles or ADC constructs could enable spatially restricted delivery of xCT inhibitors, TLR2 modulators, or nucleic acid-based therapies, broadening the therapeutic window while limiting off-target toxicity [198,199]. These considerations will be essential for the safe and effective integration of xCT-targeted agents into combinatorial regimens.

To overcome the limitations of small-molecule inhibitors, immunotherapeutic strategies have also been developed to selectively target xCT. DNA- and viral-based vaccines have been shown to induce robust anti-xCT immune responses in murine models, generating antibodies and cytotoxic T cells capable of recognizing and eliminating xCT-expressing tumor cells [62,68]. We previously demonstrated that anti-xCT DNA vaccination delays tumor growth and metastasis in TNBC models by perturbing CSC self-renewal and redox balance through the induction of anti-xCT antibodies [62]. Importantly, xCT-targeted vaccines synergize with other therapeutic modalities, including human epidermal growth factor receptor 2 (HER2)-directed therapies [71] and the p53 reactivator APR-246 [72], by engaging complementary metabolic and immunologic mechanisms. For example, combined delivery of bovine herpesvirus 4 (BoHV-4)-based viral vectors expressing HER2 and xCT antigens elicited high-titer antibodies that mediated cytotoxicity, inhibited CSC proliferation, and suppressed metastasis in breast cancer models [71]. These findings highlight the translational potential of xCT-targeted immunotherapy within rational combinatorial regimens aimed at eradicating both differentiated tumor cells and stem-like subpopulations [200]. Notably, the recently uncovered roles of xCT in OSA, where it supports redox balance, and sustains OSA cell survival and proliferation under metabolic stress [20,21], position xCT as both a metabolic vulnerability and a promising immunotherapeutic target for OSA. Evidence from other preclinical models showing that anti-xCT vaccination can enhance tumor chemosensitivity to DOXO [62] further supports the consideration of this strategy as a potential combinatorial approach to be integrated alongside standard chemotherapy in the OSA setting.

The induction of an immune response capable of impairing the malignant behavior of cancer cells through both immune-mediated and non-immune mechanisms, as suggested above, would therefore be highly desirable. Such an approach could generate long-lasting immune memory, potentially preventing tumor recurrence and metastasis. Nevertheless, the strongly immunosuppressive TME characteristic of OSA may limit vaccine efficacy. In this context, modulation of the TME through TLR2 targeting may further enhance therapeutic outcomes.

Different approaches have been proposed to target TLR2, whose dual role can mediate both anti-tumor immunity and pro-tumorigenic signaling. The use of TLR2 agonists (e.g., Pam3CSK4, PSK) as adjuvants has shown the ability to antigen presentation, DC maturation, and natural killer (NK) cell activation, thereby improving the efficacy of anticancer vaccines and mAbs. However, these agonists must be used cautiously because of potential pro-tumorigenic effects, including Treg expansion [37,73].

Conversely, our group demonstrated that the tumor-targeted inhibition of TLR2 represents a key strategy in malignancy such as breast cancer, where high TLR2 expression correlates with poor prognosis and chemoresistance. The selective TLR2 inhibitor CU-CPT22 effectively blocked DAMP-induced signaling (e.g., HMGB1 release after chemotherapy), synergizing with DOXO to suppress tumor growth, reduce CSC frequency, and remodel the TME by decreasing Tregs and MDSCs while enhancing CD8^+^ T cells and M1 macrophages [37]. Consistently, preclinical studies using genetic or antibody-mediated TLR2 blockade (e.g., OPN-301) inhibited tumorigenesis in gastric and pancreatic carcinomas, and head and neck cancers [201].

In clinical settings, TLR2 inhibitors are being investigated in combination regimens, enhancing the efficacy of chemotherapy, anti-angiogenic agents (e.g., bevacizumab), and immunotherapies, showing synergy with TLR9 agonists (CpG ODN) in melanoma models [29]. Such combinations may be particularly relevant for OSA, where effective treatments remain focused on chemotherapy.

## 8. Conclusions

The urgent need to develop novel therapies for the management of OSA patients is challenged by the intrinsic difficulty of targeting this tumor type, which is inherently resistant to treatment due to its biological properties. The identification of new therapeutic vulnerabilities to be exploited for OSA treatment offers promising opportunities to address this critical clinical challenge.

CSPG4, xCT, and TLR2 participate in multiple cancer hallmarks and influence diverse aspects of OSA biology through distinct mechanisms, contributing to OSA pathogenesis and progression. CSPG4 promotes tumor cell adhesion, proliferation, migration, and resistance to therapy through its interactions with the ECM and the regulation of key signaling pathways. xCT maintains redox balance and mediates glutamate release, promoting tumor cell survival under oxidative stress and a pro-tumorigenic microenvironment. Meanwhile, TLR2 modulates immune responses and inflammation, influencing both tumor growth and the crosstalk between cancer and immune cells. Taken together, these molecules contribute to the complex interplay among tumor, stroma, and immune components that drives OSA aggressiveness.

While the rationale for targeting CSPG4 in OSA has already been established, further investigation into the roles of xCT and TLR2 is warranted. Such studies could reveal previously unexplored mechanisms contributing to osteosarcomagenesis and disease progression. Given that these three molecules are implicated in partially overlapping yet distinct aspects of tumor progression, a combinatorial therapeutic strategy that simultaneously targets CSPG4, xCT, and TLR2 may represent a novel and more effective approach to disrupt multiple tumor-supportive mechanisms. This combined therapy could potentially overcome resistance to single-agent treatments and improve patient outcomes. Future studies should aim to elucidate the molecular crosstalk among these pathways and develop integrated therapeutic regimens suitable for clinical translation.

Moreover, validating the roles of these three molecules, individually or in combination, across different OSA subtypes and in relation to standard of care treatment responses could facilitate patient stratification and guide the design of novel, precision-based therapeutic interventions. A rational, evidence-based, multi-targeting strategy, to be used alone or in synergy with the standard of care, may ultimately lead to safer and more effective multimodal treatments capable of overcoming immune resistance and improving clinical outcomes for OSA patients, for whom there is a paucity of effective therapeutic options.

## Data Availability

No new data were created or analyzed in this study. Data sharing is not applicable to this article.

## Abbreviations

The following abbreviations are used in this manuscript:

ABCB1	ATP-Binding Cassette Subfamily B Member 1 (P-glycoprotein)
ABCC1	ATP-Binding Cassette Subfamily C Member 1 (MRP1)
ATF4	Activating Transcription Factor 4
B7-H3	B7 Homolog 3
Bregs	B Regulatory Cells
cAMP	cyclic Adenosine Monophosphate
CARs	Chimeric Antigen Receptors
CDDP	Cisplatin
CEP	Carboxyethylpyrrole
CREB	cAMP Response Element-Binding protein
CS	Chondroitin-Sulfate
CSCs	Cancer stem cells
CSPG4	Chondroitin Sulfate Proteoglycan 4
DAMPs	Damage-Associated Molecular Patterns
DCs	Dendritic Cell
DOXO	Doxorubicin
ECM	Extracellular Matrix
EGFR	Epidermal Growth Factor Receptor
EMT	Epithelial-to-Mesenchymal Transition
ERK1/2	Extracellular Signal-Regulated Kinases 1 and 2
ERO1α	Endoplasmic Reticulum Oxidoreductin 1-alpha
EV	Extracellular Vesicle
FAK	Focal Adhesion Kinase
FGF-2	Fibroblast Growth Factor 2
GSH	Glutathione
HER2	Human Epidermal Growth Factor Receptor 2
HIF1α	Hypoxia-Inducible Factor 1-alpha
HMGB1	High-Mobility Group Box 1
HSP90	Heat Shock Protein 90
ICIs	Immune Checkpoint Inhibitors
IFN	Interferon
IFO	Ifosfamide
IGF-1R	Insulin-like Growth Factor 1 Receptor
IGFBPs	Insulin-like Growth Factor Binding Proteins
IL-10	Inteleukin-10
IL-21	Interleukin-21
IL1RAP	IL-1 Receptor Accessory Protein
IRFs	Interferon Regulatory Factors
mAbs	Monoclonal Antibodies
MAPK	Mitogen-Activated Protein Kinase
MDSCs	Myeloid Derived Suppressor Cells
mGluR	Metabotropic glutamate receptor
MLX	MAX-like Protein X
MMP	Metalloproteinase
mTOR	Mechanistic Target of Rapamycin
MTX	Methotrexate
MUC1	Mucin 1
MyD88	Myeloid Differentiation Primary Response 88
NF-κB	Nuclear Factor kappa-light-chain-enhancer of activated B cells
NRF2	Nuclear Factor Erythroid 2-Related Factor 2
OSA	Osteosarcoma
PAMPs	Pathogen-Associated Molecular Patterns
PD-1	Programmed Cell Death Protein-1
PD-L1	Programmed Death Ligand-1
PDGF	Platelet-Derived Growth Factor
PDGFRα	Platelet-Derived Growth Factor Receptor alpha
PI3K	Phosphoinositide 3-Kinase
PKA	Protein Kinase A
PKCα	Protein Kinase C alpha
PRRs	Pattern Recognition Receptors
RAGE	Receptor for Advanced Glycation End-products
ROS	Reactive Oxygen Species
SAS	Sulfasalazine
STAT3	Signal Transducer and Activator of Transcription 3
TAAs	Tumor-Associated Antigens
TCR	T-cell receptor
TLR2	Toll-like Receptor 2
TME	Tumor Microenvironment
Tregs	T Regulatory Cells
VEGF	Vascular Endothelial Growth Factor
ZAP70	Zeta-chain Associated Protein Kinase 70

## References

[1] Beird H.C., Bielack S.S., Flanagan A.M., Gill J., Heymann D., Janeway K.A., Livingston J.A., Roberts R.D., Strauss S.J., Gorlick R. (2022). Osteosarcoma. Nat. Rev. Dis. Primers.

[2] Kim C., Davis L.E., Albert C.M., Samuels B., Roberts J.L., Wagner M.J. (2023). Osteosarcoma in Pediatric and Adult Populations: Are Adults Just Big Kids?. Cancers.

[3] Banjara R., Kumar V.S., Khan S.A., Majeed A., Poudel R.R., Kanwat H., Thapa S. (2021). Relationship between height and osteosarcoma at the time of diagnosis in the Indian population: A retrospective study. J. Clin. Orthop. Trauma.

[4] Sheng G., Gao Y., Yang Y., Wu H. (2021). Osteosarcoma and Metastasis. Front. Oncol..

[5] de Azevedo J.W.V., de Medeiros Fernandes T.A.A., Fernandes J.V., de Azevedo J.C.V., Lanza D.C.F., Bezerra C.M., Andrade V.S., de Araujo J.M.G., Fernandes J.V. (2020). Biology and pathogenesis of human osteosarcoma. Oncol. Lett..

[6] Dasari S., Tchounwou P.B. (2014). Cisplatin in cancer therapy: Molecular mechanisms of action. Eur. J. Pharmacol..

[7] Marchandet L., Lallier M., Charrier C., Baud’huin M., Ory B., Lamoureux F. (2021). Mechanisms of Resistance to Conventional Therapies for Osteosarcoma. Cancers.

[8] Patel T.D., Grimm S.L., Kanchi R.S., Gandhi T., Koirala A., Yustein J.T., Coarfa C. (2024). Identification of an early survival prognostic gene signature for localized osteosarcoma patients. Sci. Rep..

[9] Fagioli F., Biasin E., Mereuta O.M., Muraro M., Luksch R., Ferrari S., Aglietta M., Madon E. (2008). Poor prognosis osteosarcoma: New therapeutic approach. Bone Marrow Transpl..

[10] Garcia-Ortega D.Y., Cabrera-Nieto S.A., Caro-Sanchez H.S., Cruz-Ramos M. (2022). An overview of resistance to chemotherapy in osteosarcoma and future perspectives. Cancer Drug Resist..

[11] Tarone L., Giacobino D., Camerino M., Maniscalco L., Iussich S., Parisi L., Giovannini G., Dentini A., Bolli E., Quaglino E. (2023). A chimeric human/dog-DNA vaccine against CSPG4 induces immunity with therapeutic potential in comparative preclinical models of osteosarcoma. Mol. Ther..

[12] Riccardo F., Tarone L., Iussich S., Giacobino D., Arigoni M., Sammartano F., Morello E., Martano M., Gattino F., Maria R. (2019). Identification of CSPG4 as a promising target for translational combinatorial approaches in osteosarcoma. Ther. Adv. Med. Oncol..

[13] Rolih V., Barutello G., Iussich S., De Maria R., Quaglino E., Buracco P., Cavallo F., Riccardo F. (2017). CSPG4: A prototype oncoantigen for translational immunotherapy studies. J. Transl. Med..

[14] Price M.A., Colvin Wanshura L.E., Yang J., Carlson J., Xiang B., Li G., Ferrone S., Dudek A.Z., Turley E.A., McCarthy J.B. (2011). CSPG4, a potential therapeutic target, facilitates malignant progression of melanoma. Pigment Cell Melanoma Res..

[15] Kurokawa T., Imai K. (2024). Chondroitin sulfate proteoglycan 4: An attractive target for antibody-based immunotherapy. Proc. Jpn. Acad. Ser. B Phys. Biol. Sci..

[16] Uno K., Koya Y., Yoshihara M., Iyoshi S., Kitami K., Sugiyama M., Miyamoto E., Mogi K., Fujimoto H., Yamakita Y. (2024). Chondroitin Sulfate Proteoglycan 4 Provides New Treatment Approach to Preventing Peritoneal Dissemination in Ovarian Cancer. Int. J. Mol. Sci..

[17] Niibori-Nambu A., Yamasaki Y., Kobayashi D., Angata K., Kuno A., Panawan O., Silsirivanit A., Narimatsu H., Araki N. (2024). Chondroitin sulfate modification of CSPG4 regulates the maintenance and differentiation of glioma-initiating cells via integrin-associated signaling. J. Biol. Chem..

[18] Chen X., Habib S., Alexandru M., Chauhan J., Evan T., Troka J.M., Rahimi A., Esapa B., Tull T.J., Ng W.Z. (2024). Chondroitin Sulfate Proteoglycan 4 (CSPG4) as an Emerging Target for Immunotherapy to Treat Melanoma. Cancers.

[19] Tarone L., Mareschi K., Tirtei E., Giacobino D., Camerino M., Buracco P., Morello E., Cavallo F., Riccardo F. (2022). Improving Osteosarcoma Treatment: Comparative Oncology in Action. Life.

[20] Guo W., Wang X., Lu B., Yu J., Xu M., Huang R., Cheng M., Yang M., Zhao W., Zou C. (2023). Super-enhancer-driven MLX mediates redox balance maintenance via SLC7A11 in osteosarcoma. Cell Death Dis..

[21] Zou Q., Zhou X., Lai J., Zhou H., Su J., Zhang Z., Zhuang X., Liu L., Yuan R., Li S. (2025). Targeting p62 by sulforaphane promotes autolysosomal degradation of SLC7A11, inducing ferroptosis for osteosarcoma treatment. Redox Biol..

[22] Wen R.J., Dong X., Zhuang H.W., Pang F.X., Ding S.C., Li N., Mai Y.X., Zhou S.T., Wang J.Y., Zhang J.F. (2023). Baicalin induces ferroptosis in osteosarcomas through a novel Nrf2/xCT/GPX4 regulatory axis. Phytomedicine.

[23] Huang R., Chu D., Shi J., Xu R., Wang K. (2024). Shikonin suppresses proliferation of osteosarcoma cells by inducing ferroptosis through promoting Nrf2 ubiquitination and inhibiting the xCT/GPX4 regulatory axis. Front. Pharmacol..

[24] Qin Q., Zhang H., Lai M., Wei J., Qian J., Chen X., Wang X., Wang Y. (2025). Sulfasalazine induces ferroptosis in osteosarcomas by regulating Nrf2/SLC7A11/GPX4 signaling axis. Sci. Rep..

[25] Wang P., Xiao J., Zeng J., Yang F., Lin M., Liang T., Liu H., Zhan H. (2025). PSAT1 inhibits ferroptosis in osteosarcoma cells by activating the Xct/GPX4 signaling axis. Sci. Rep..

[26] Jing Y., Liang H., Zhang Y., Cleveland J., Yan J., Zhang D. (2015). Up-regulation of Toll-like Receptor 9 in Osteosarcoma. Anticancer Res..

[27] Magri J., Gasparetto A., Conti L., Calautti E., Cossu C., Ruiu R., Barutello G., Cavallo F. (2021). Tumor-Associated Antigen xCT and Mutant-p53 as Molecular Targets for New Combinatorial Antitumor Strategies. Cells.

[28] Landuzzi L., Ruzzi F., Pellegrini E., Lollini P.L., Scotlandi K., Manara M.C. (2024). IL-1 Family Members in Bone Sarcomas. Cells.

[29] Di Lorenzo A., Bolli E., Tarone L., Cavallo F., Conti L. (2020). Toll-Like Receptor 2 at the Crossroad between Cancer Cells, the Immune System, and the Microbiota. Int. J. Mol. Sci..

[30] Sun Y., Zhang C., Fang Q., Zhang W., Liu W. (2023). Abnormal signal pathways and tumor heterogeneity in osteosarcoma. J. Transl. Med..

[31] Tamburini E., Dallatomasina A., Quartararo J., Cortelazzi B., Mangieri D., Lazzaretti M., Perris R. (2019). Structural deciphering of the NG2/CSPG4 proteoglycan multifunctionality. FASEB J..

[32] Ilieva K.M., Cheung A., Mele S., Chiaruttini G., Crescioli S., Griffin M., Nakamura M., Spicer J.F., Tsoka S., Lacy K.E. (2017). Chondroitin Sulfate Proteoglycan 4 and Its Potential As an Antibody Immunotherapy Target across Different Tumor Types. Front. Immunol..

[33] Liu X., Du S., Wang S., Ye K. (2022). Ferroptosis in osteosarcoma: A promising future. Front. Oncol..

[34] Lemaitre B. (2004). The road to Toll. Nat. Rev. Immunol..

[35] Park D.W., Lyu J.H., Kim J.S., Chin H., Bae Y.S., Baek S.H. (2013). Role of JAK2-STAT3 in TLR2-mediated tissue factor expression. J. Cell. Biochem..

[36] Conti L., Lanzardo S., Arigoni M., Antonazzo R., Radaelli E., Cantarella D., Calogero R.A., Cavallo F. (2013). The noninflammatory role of high mobility group box 1/Toll-like receptor 2 axis in the self-renewal of mammary cancer stem cells. FASEB J..

[37] Di Lorenzo A., Bolli E., Ruiu R., Ferrauto G., Di Gregorio E., Avalle L., Savino A., Poggio P., Merighi I.F., Riccardo F. (2022). Toll-like receptor 2 promotes breast cancer progression and resistance to chemotherapy. Oncoimmunology.

[38] Wang Y.F., Feng J.Y., Zhao L.N., Zhao M., Wei X.F., Geng Y., Yuan H.F., Hou C.Y., Zhang H.H., Wang G.W. (2023). Aspirin triggers ferroptosis in hepatocellular carcinoma cells through restricting NF-κB p65-activated SLC7A11 transcription. Acta Pharmacol. Sin..

[39] Linher-Melville K., Singh G. (2017). The complex roles of STAT3 and STAT5 in maintaining redox balance: Lessons from STAT-mediated xCT expression in cancer cells. Mol. Cell. Endocrinol..

[40] Lee S.B., Sellers B.N., DeNicola G.M. (2018). The Regulation of NRF2 by Nutrient-Responsive Signaling and Its Role in Anabolic Cancer Metabolism. Antioxid. Redox Signal..

[41] Groft S.G., Nagy N., Boom W.H., Harding C.V. (2020). Toll-Like Receptor 2-Tpl2-Dependent ERK Signaling Drives Inverse Interleukin 12 Regulation in Dendritic Cells and Macrophages. Infect. Immun..

[42] Pathria G., Ronai Z.A. (2021). Harnessing the Co-vulnerabilities of Amino Acid-Restricted Cancers. Cell Metab..

[43] Zhu Y., Yang L., Yu Y., Xiong Y., Xiao P., Fu X., Luo X. (2024). Hydroxysafflor yellow A induced ferroptosis of Osteosarcoma cancer cells by HIF-1α/HK2 and SLC7A11 pathway. Oncol. Res..

[44] Keleg S., Titov A., Heller A., Giese T., Tjaden C., Ahmad S.S., Gaida M.M., Bauer A.S., Werner J., Giese N.A. (2014). Chondroitin sulfate proteoglycan CSPG4 as a novel hypoxia-sensitive marker in pancreatic tumors. PLoS ONE.

[45] Kuhlicke J., Frick J.S., Morote-Garcia J.C., Rosenberger P., Eltzschig H.K. (2007). Hypoxia inducible factor (HIF)-1 coordinates induction of Toll-like receptors TLR2 and TLR6 during hypoxia. PLoS ONE.

[46] Geldres C., Savoldo B., Hoyos V., Caruana I., Zhang M., Yvon E., Del Vecchio M., Creighton C.J., Ittmann M., Ferrone S. (2014). T lymphocytes redirected against the chondroitin sulfate proteoglycan-4 control the growth of multiple solid tumors both in vitro and in vivo. Clin. Cancer Res..

[47] Yang J., Liao Q., Price M., Moriarity B., Wolf N., Felices M., Miller J.S., Geller M.A., Bendzick L., Hopps R. (2022). Chondroitin sulfate proteoglycan 4, a targetable oncoantigen that promotes ovarian cancer growth, invasion, cisplatin resistance and spheroid formation. Transl. Oncol..

[48] Schiffer D., Mellai M., Boldorini R., Bisogno I., Grifoni S., Corona C., Bertero L., Cassoni P., Casalone C., Annovazzi L. (2018). The Significance of Chondroitin Sulfate Proteoglycan 4 (CSPG4) in Human Gliomas. Int. J. Mol. Sci..

[49] Wang X., Wang Y., Yu L., Sakakura K., Visus C., Schwab J.H., Ferrone C.R., Favoino E., Koya Y., Campoli M.R. (2010). CSPG4 in cancer: Multiple roles. Curr. Mol. Med..

[50] Rivera Z., Ferrone S., Wang X., Jube S., Yang H., Pass H.I., Kanodia S., Gaudino G., Carbone M. (2012). CSPG4 as a target of antibody-based immunotherapy for malignant mesothelioma. Clin. Cancer Res..

[51] Chen K., Yong J., Zauner R., Wally V., Whitelock J., Sajinovic M., Kopecki Z., Liang K., Scott K.F., Mellick A.S. (2022). Chondroitin Sulfate Proteoglycan 4 as a Marker for Aggressive Squamous Cell Carcinoma. Cancers.

[52] Mellai M., Casalone C., Corona C., Crociara P., Favole A., Cassoni P., Schiffer D., Boldorini R. (2020). Chondroitin Sulphate Proteoglycans in the Tumour Microenvironment. Adv. Exp. Med. Biol..

[53] Nicolosi P.A., Dallatomasina A., Perris R. (2015). Theranostic impact of NG2/CSPG4 proteoglycan in cancer. Theranostics.

[54] Chekenya M., Krakstad C., Svendsen A., Netland I.A., Staalesen V., Tysnes B.B., Selheim F., Wang J., Sakariassen P.O., Sandal T. (2008). The progenitor cell marker NG2/MPG promotes chemoresistance by activation of integrin-dependent PI3K/Akt signaling. Oncogene.

[55] Wilson B.S., Imai K., Natali P.G., Ferrone S. (1981). Distribution and molecular characterization of a cell-surface and a cytoplasmic antigen detectable in human melanoma cells with monoclonal antibodies. Int. J. Cancer.

[56] Jiang Y., Sun M. (2024). SLC7A11: The Achilles heel of tumor?. Front. Immunol..

[57] Bhutia Y.D., Babu E., Ramachandran S., Ganapathy V. (2015). Amino Acid transporters in cancer and their relevance to “glutamine addiction”: Novel targets for the design of a new class of anticancer drugs. Cancer Res..

[58] Ostrowski M., Carmo N.B., Krumeich S., Fanget I., Raposo G., Savina A., Moita C.F., Schauer K., Hume A.N., Freitas R.P. (2010). Rab27a and Rab27b control different steps of the exosome secretion pathway. Nat. Cell Biol..

[59] Chen R.S., Song Y.M., Zhou Z.Y., Tong T., Li Y., Fu M., Guo X.L., Dong L.J., He X., Qiao H.X. (2009). Disruption of xCT inhibits cancer cell metastasis via the caveolin-1/β-catenin pathway. Oncogene.

[60] Dornier E., Rabas N., Mitchell L., Novo D., Dhayade S., Marco S., Mackay G., Sumpton D., Pallares M., Nixon C. (2017). Glutaminolysis drives membrane trafficking to promote invasiveness of breast cancer cells. Nat. Commun..

[61] Wang S.F., Chen M.S., Chou Y.C., Ueng Y.F., Yin P.H., Yeh T.S., Lee H.C. (2016). Mitochondrial dysfunction enhances cisplatin resistance in human gastric cancer cells via the ROS-activated GCN2-eIF2α-ATF4-xCT pathway. Oncotarget.

[62] Lanzardo S., Conti L., Rooke R., Ruiu R., Accart N., Bolli E., Arigoni M., Macagno M., Barrera G., Pizzimenti S. (2016). Immunotargeting of Antigen xCT Attenuates Stem-like Cell Behavior and Metastatic Progression in Breast Cancer. Cancer Res..

[63] Yoshikawa M., Tsuchihashi K., Ishimoto T., Yae T., Motohara T., Sugihara E., Onishi N., Masuko T., Yoshizawa K., Kawashiri S. (2013). xCT inhibition depletes CD44v-expressing tumor cells that are resistant to EGFR-targeted therapy in head and neck squamous cell carcinoma. Cancer Res..

[64] Nagano O., Okazaki S., Saya H. (2013). Redox regulation in stem-like cancer cells by CD44 variant isoforms. Oncogene.

[65] Hasegawa M., Takahashi H., Rajabi H., Alam M., Suzuki Y., Yin L., Tagde A., Maeda T., Hiraki M., Sukhatme V.P. (2016). Functional interactions of the cystine/glutamate antiporter, CD44v and MUC1-C oncoprotein in triple-negative breast cancer cells. Oncotarget.

[66] Zhang Y., Park M., Ghoda L.Y., Zhao D., Valerio M., Nafie E., Gonzalez A., Ly K., Parcutela B., Choi H. (2024). IL1RAP-specific T cell engager depletes acute myeloid leukemia stem cells. J. Hematol. Oncol..

[67] Carroll P.A., Freie B.W., Cheng P.F., Kasinathan S., Gu H., Hedrich T., Dowdle J.A., Venkataramani V., Ramani V., Wu X. (2021). The glucose-sensing transcription factor MLX balances metabolism and stress to suppress apoptosis and maintain spermatogenesis. PLoS Biol..

[68] Bolli E., O’Rourke J.P., Conti L., Lanzardo S., Rolih V., Christen J.M., Barutello G., Forni M., Pericle F., Cavallo F. (2018). A Virus-Like-Particle immunotherapy targeting Epitope-Specific anti-xCT expressed on cancer stem cell inhibits the progression of metastatic cancer in vivo. Oncoimmunology.

[69] Ruiu R., Rolih V., Bolli E., Barutello G., Riccardo F., Quaglino E., Merighi I.F., Pericle F., Donofrio G., Cavallo F. (2019). Fighting breast cancer stem cells through the immune-targeting of the xCT cystine-glutamate antiporter. Cancer Immunol. Immunother..

[70] Donofrio G., Tebaldi G., Lanzardo S., Ruiu R., Bolli E., Ballatore A., Rolih V., Macchi F., Conti L., Cavallo F. (2018). Bovine herpesvirus 4-based vector delivering the full length xCT DNA efficiently protects mice from mammary cancer metastases by targeting cancer stem cells. Oncoimmunology.

[71] Conti L., Bolli E., Di Lorenzo A., Franceschi V., Macchi F., Riccardo F., Ruiu R., Russo L., Quaglino E., Donofrio G. (2020). Immunotargeting of the xCT Cystine/Glutamate Antiporter Potentiates the Efficacy of HER2-Targeted Immunotherapies in Breast Cancer. Cancer Immunol. Res..

[72] Barutello G., Di Lorenzo A., Gasparetto A., Galiazzi C., Bolli E., Conti L., Cavallo F. (2022). Immunotherapy against the Cystine/Glutamate Antiporter xCT Improves the Efficacy of APR-246 in Preclinical Breast Cancer Models. Biomedicines.

[73] Cossu C., Di Lorenzo A., Fiorilla I., Todesco A.M., Audrito V., Conti L. (2023). The Role of the Toll-like Receptor 2 and the cGAS-STING Pathways in Breast Cancer: Friends or Foes?. Int. J. Mol. Sci..

[74] Kuo A.H., Scheeren F.A. (2014). Cell-intrinsic TLR2/MyD88 pathway in breast and colon cancer. Cell Cycle.

[75] McCoy M.G., Nascimento D.W., Veleeparambil M., Murtazina R., Gao D., Tkachenko S., Podrez E., Byzova T.V. (2021). Endothelial TLR2 promotes proangiogenic immune cell recruitment and tumor angiogenesis. Sci. Signal..

[76] Ko J.H., Lee H.J., Yoon C.H., Choi Y.R., Ryu J.S., Oh J.Y. (2024). Activation of Toll-like receptor 2 promotes mesenchymal stem/stromal cell-mediated immunoregulation and angiostasis through AKR1C1. Theranostics.

[77] Lundy J., Gearing L.J., Gao H., West A.C., McLeod L., Deswaerte V., Yu L., Porazinski S., Pajic M., Hertzog P.J. (2021). TLR2 activation promotes tumour growth and associates with patient survival and chemotherapy response in pancreatic ductal adenocarcinoma. Oncogene.

[78] Tirtei E., Campello A., Sciannameo V., Asaftei S.D., Meazza C., Sironi G., Longhi A., Ibrahim T., Tamburini A., Coccoli L. (2024). Prolonged 14-day continuous infusion of high-dose ifosfamide for patients with relapsed and refractory high-grade osteosarcoma: A retrospective multicentre cohort study. BMC Cancer.

[79] Harris M.A., Hawkins C.J. (2022). Recent and Ongoing Research into Metastatic Osteosarcoma Treatments. Int. J. Mol. Sci..

[80] Wu C.C., Livingston J.A. (2020). Genomics and the Immune Landscape of Osteosarcoma. Adv. Exp. Med. Biol..

[81] Meijer D.M., Ruano D., Briaire-de Bruijn I.H., Wijers-Koster P.M., van de Sande M.A.J., Gelderblom H., Cleton-Jansen A.M., de Miranda N., Kuijjer M.L., Bovee J. (2024). The Variable Genomic Landscape During Osteosarcoma Progression: Insights From a Longitudinal WGS Analysis. Genes Chromosomes Cancer.

[82] Rajan S., Zaccaria S., Cannon M.V., Cam M., Gross A.C., Raphael B.J., Roberts R.D. (2023). Structurally Complex Osteosarcoma Genomes Exhibit Limited Heterogeneity within Individual Tumors and across Evolutionary Time. Cancer Res. Commun..

[83] Xie L., Yang Y., Guo W., Che D., Xu J., Sun X., Liu K., Ren T., Liu X., Yang Y. (2020). The Clinical Implications of Tumor Mutational Burden in Osteosarcoma. Front. Oncol..

[84] Chen Z., Guo J., Zhang K., Guo Y. (2016). TP53 Mutations and Survival in Osteosarcoma Patients: A Meta-Analysis of Published Data. Dis. Markers.

[85] Jiang Y., Wang J., Sun M., Zuo D., Wang H., Shen J., Jiang W., Mu H., Ma X., Yin F. (2022). Multi-omics analysis identifies osteosarcoma subtypes with distinct prognosis indicating stratified treatment. Nat. Commun..

[86] Zoumpoulidou G., Alvarez-Mendoza C., Mancusi C., Ahmed R.M., Denman M., Steele C.D., Tarabichi M., Roy E., Davies L.R., Manji J. (2021). Therapeutic vulnerability to PARP1,2 inhibition in RB1-mutant osteosarcoma. Nat. Commun..

[87] Czarnecka A.M., Synoradzki K., Firlej W., Bartnik E., Sobczuk P., Fiedorowicz M., Grieb P., Rutkowski P. (2020). Molecular Biology of Osteosarcoma. Cancers.

[88] Zhang N., Jing Z., Song J., Liang Q., Xu Y., Xu Z., Wen L., Wei P. (2025). Discovery of Drugs Targeting Mutant p53 and Progress in Nano-Enabled Therapeutic Strategy for p53-Mutated Cancers. Biomolecules.

[89] Zhang W., Liu Y., Luo Y., Xu J., Zhang B., Feng P., Guo C., Wang Y., Huang Z., Kong Q. (2025). Reactivating P53 to treat osteosarcoma: A tetrahedral framework nucleic acids-based approach. Int. J. Biol. Macromol..

[90] Linn P., Kohno S., Sheng J., Kulathunga N., Yu H., Zhang Z., Voon D., Watanabe Y., Takahashi C. (2021). Targeting RB1 Loss in Cancers. Cancers.

[91] Shen J., Wang Q., Mao Y., Gao W., Duan S. (2023). Targeting the p53 signaling pathway in cancers: Molecular mechanisms and clinical studies. MedComm.

[92] Hanahan D., Weinberg R.A. (2011). Hallmarks of cancer: The next generation. Cell.

[93] Chen C., Shi Q., Xu J., Ren T., Huang Y., Guo W. (2022). Current progress and open challenges for applying tyrosine kinase inhibitors in osteosarcoma. Cell Death Discov..

[94] Tian Z., Niu X., Yao W. (2020). Receptor Tyrosine Kinases in Osteosarcoma Treatment: Which Is the Key Target?. Front. Oncol..

[95] Honoki K., Tsujiuchi T., Kishi S., Kuniyasu H. (2024). Revisiting ‘Hallmarks of Cancer’ In Sarcomas. J. Cancer.

[96] Assi A., Farhat M., Hachem M.C.R., Zalaquett Z., Aoun M., Daher M., Sebaaly A., Kourie H.R. (2023). Tyrosine kinase inhibitors in osteosarcoma: Adapting treatment strategiesa. J. Bone Oncol..

[97] Wang J., Svendsen A., Kmiecik J., Immervoll H., Skaftnesmo K.O., Planaguma J., Reed R.K., Bjerkvig R., Miletic H., Enger P.O. (2011). Targeting the NG2/CSPG4 proteoglycan retards tumour growth and angiogenesis in preclinical models of GBM and melanoma. PLoS ONE.

[98] Wang X., Osada T., Wang Y., Yu L., Sakakura K., Katayama A., McCarthy J.B., Brufsky A., Chivukula M., Khoury T. (2010). CSPG4 protein as a new target for the antibody-based immunotherapy of triple-negative breast cancer. J. Natl. Cancer Inst..

[99] Hsu S.C., Nadesan P., Puviindran V., Stallcup W.B., Kirsch D.G., Alman B.A. (2018). Effects of chondroitin sulfate proteoglycan 4 (NG2/CSPG4) on soft-tissue sarcoma growth depend on tumor developmental stage. J. Biol. Chem..

[100] Kuijjer M.L., Rydbeck H., Kresse S.H., Buddingh E.P., Lid A.B., Roelofs H., Burger H., Myklebost O., Hogendoorn P.C., Meza-Zepeda L.A. (2012). Identification of osteosarcoma driver genes by integrative analysis of copy number and gene expression data. Genes Chromosomes Cancer.

[101] Kuijjer M.L., Peterse E.F., van den Akker B.E., Briaire-de Bruijn I.H., Serra M., Meza-Zepeda L.A., Myklebost O., Hassan A.B., Hogendoorn P.C., Cleton-Jansen A.M. (2013). IR/IGF1R signaling as potential target for treatment of high-grade osteosarcoma. BMC Cancer.

[102] Chow L., Ammons D., Dow S.W. (2025). Role of companion dogs in cancer immunotherapy development. J. Immunol..

[103] Simpson S., Rizvanov A.A., Jeyapalan J.N., de Brot S., Rutland C.S. (2022). Canine osteosarcoma in comparative oncology: Molecular mechanisms through to treatment discovery. Front. Vet. Sci..

[104] Mannheimer J.D., Tawa G., Gerhold D., Braisted J., Sayers C.M., McEachron T.A., Meltzer P., Mazcko C., Beck J.A., LeBlanc A.K. (2023). Transcriptional profiling of canine osteosarcoma identifies prognostic gene expression signatures with translational value for humans. Commun. Biol..

[105] Morello E., Martano M., Buracco P. (2011). Biology, diagnosis and treatment of canine appendicular osteosarcoma: Similarities and differences with human osteosarcoma. Vet. J..

[106] Polton G., Borrego J.F., Clemente-Vicario F., Clifford C.A., Jagielski D., Kessler M., Kobayashi T., Lanore D., Queiroga F.L., Rodrigues L. (2025). Osteosarcoma of the appendicular skeleton in dogs: Consensus and guidelines. Front. Vet. Sci..

[107] Liu Y.D., Ji C.B., Li S.B., Yan F., Gu Q.S., Balic J.J., Yu L., Li J.K. (2018). Toll-like receptor 2 stimulation promotes colorectal cancer cell growth via PI3K/Akt and NF-κB signaling pathways. Int. Immunopharmacol..

[108] Zhang W., Liu W., Hu X. (2023). Robinin inhibits pancreatic cancer cell proliferation, EMT and inflammation via regulating TLR2-PI3k-AKT signaling pathway. Cancer Cell Int..

[109] Liu B., Yan S., Jia Y., Ma J., Wu S., Xu Y., Shang M., Mao A. (2016). TLR2 promotes human intrahepatic cholangiocarcinoma cell migration and invasion by modulating NF-κB pathway-mediated inflammatory responses. FEBS J..

[110] Koppula P., Zhuang L., Gan B. (2021). Cystine transporter SLC7A11/xCT in cancer: Ferroptosis, nutrient dependency, and cancer therapy. Protein Cell.

[111] Li B., Ming H., Qin S., Nice E.C., Dong J., Du Z., Huang C. (2025). Redox regulation: Mechanisms, biology and therapeutic targets in diseases. Signal Transduct. Target. Ther..

[112] Liu X., Zhang Y., Zhuang L., Olszewski K., Gan B. (2021). NADPH debt drives redox bankruptcy: SLC7A11/xCT-mediated cystine uptake as a double-edged sword in cellular redox regulation. Genes Dis..

[113] Su Z., Liu Y., Wang L., Gu W. (2025). Regulation of SLC7A11 as an unconventional checkpoint in tumorigenesis through ferroptosis. Genes Dis..

[114] Koppula P., Zhang Y., Zhuang L., Gan B. (2018). Amino acid transporter SLC7A11/xCT at the crossroads of regulating redox homeostasis and nutrient dependency of cancer. Cancer Commun..

[115] Prickett T.D., Samuels Y. (2012). Molecular pathways: Dysregulated glutamatergic signaling pathways in cancer. Clin. Cancer Res..

[116] Jyotsana N., Ta K.T., DelGiorno K.E. (2022). The Role of Cystine/Glutamate Antiporter SLC7A11/xCT in the Pathophysiology of Cancer. Front. Oncol..

[117] Sato M., Kusumi R., Hamashima S., Kobayashi S., Sasaki S., Komiyama Y., Izumikawa T., Conrad M., Bannai S., Sato H. (2018). The ferroptosis inducer erastin irreversibly inhibits system x_c_− and synergizes with cisplatin to increase cisplatin’s cytotoxicity in cancer cells. Sci. Rep..

[118] Polewski M.D., Reveron-Thornton R.F., Cherryholmes G.A., Marinov G.K., Cassady K., Aboody K.S. (2016). Increased Expression of System xc- in Glioblastoma Confers an Altered Metabolic State and Temozolomide Resistance. Mol. Cancer Res..

[119] Ge C., Cao B., Feng D., Zhou F., Zhang J., Yang N., Feng S., Wang G., Aa J. (2017). The down-regulation of SLC7A11 enhances ROS induced P-gp over-expression and drug resistance in MCF-7 breast cancer cells. Sci. Rep..

[120] Yang Q., Li K., Huang X., Zhao C., Mei Y., Li X., Jiao L., Yang H. (2020). lncRNA *SLC7A11-AS1* Promotes Chemoresistance by Blocking SCF^β-TRCP^-Mediated Degradation of NRF2 in Pancreatic Cancer. Mol. Ther. Nucleic Acids.

[121] Starheim K.K., Holien T., Misund K., Johansson I., Baranowska K.A., Sponaas A.M., Hella H., Buene G., Waage A., Sundan A. (2016). Intracellular glutathione determines bortezomib cytotoxicity in multiple myeloma cells. Blood Cancer J..

[122] Liu J., Xia X., Huang P. (2020). xCT: A Critical Molecule That Links Cancer Metabolism to Redox Signaling. Mol. Ther..

[123] Cesar-Razquin A., Girardi E., Yang M., Brehme M., Saez-Rodriguez J., Superti-Furga G. (2018). In silico Prioritization of Transporter-Drug Relationships From Drug Sensitivity Screens. Front. Pharmacol..

[124] Girardi E., Cesar-Razquin A., Lindinger S., Papakostas K., Konecka J., Hemmerich J., Kickinger S., Kartnig F., Gurtl B., Klavins K. (2020). A widespread role for SLC transmembrane transporters in resistance to cytotoxic drugs. Nat. Chem. Biol..

[125] Wolf G., Craigon C., Teoh S.T., Essletzbichler P., Onstein S., Cassidy D., Uijttewaal E.C.H., Dvorak V., Cao Y., Bensimon A. (2025). The efflux pump ABCC1/MRP1 constitutively restricts PROTAC sensitivity in cancer cells. Cell Chem. Biol..

[126] Zhang L., Shi H., Chen H., Gong A., Liu Y., Song L., Xu X., You T., Fan X., Wang D. (2019). Dedifferentiation process driven by radiotherapy-induced HMGB1/TLR2/YAP/HIF-1α signaling enhances pancreatic cancer stemness. Cell Death Dis..

[127] Wang D.Y., Wu Y.N., Huang J.Q., Wang W., Xu M., Jia J.P., Han G., Mao B.B., Bi W.Z. (2016). Hippo/YAP signaling pathway is involved in osteosarcoma chemoresistance. Chin. J. Cancer.

[128] Wang S., Zhang Y. (2020). HMGB1 in inflammation and cancer. J. Hematol. Oncol..

[129] Chen J., He J., Yang Y., Jiang J. (2018). An analysis of the expression and function of myeloid differentiation factor 88 in human osteosarcoma. Oncol. Lett..

[130] Zhang M., Zhang X. (2015). Association of MMP-2 expression and prognosis in osteosarcoma patients. Int. J. Clin. Exp. Pathol..

[131] Cui J., Dean D., Hornicek F.J., Chen Z., Duan Z. (2020). The role of extracelluar matrix in osteosarcoma progression and metastasis. J. Exp. Clin. Cancer Res..

[132] Zhou J., Liu T., Wang W. (2018). Prognostic significance of matrix metalloproteinase 9 expression in osteosarcoma: A meta-analysis of 16 studies. Medicine.

[133] Doppelt-Flikshtain O., Asbi T., Younis A., Ginesin O., Cohen Z., Tamari T., Berg T., Yanovich C., Aran D., Zohar Y. (2024). Inhibition of osteosarcoma metastasis in vivo by targeted downregulation of MMP1 and MMP9. Matrix Biol..

[134] Ruiu R., Cossu C., Iacoviello A., Conti L., Bolli E., Ponzone L., Magri J., Rumandla A., Calautti E., Cavallo F. (2023). Cystine/glutamate antiporter xCT deficiency reduces metastasis without impairing immune system function in breast cancer mouse models. J. Exp. Clin. Cancer Res..

[135] Chen M., Jiang Y., Sun Y. (2021). KDM4A-mediated histone demethylation of SLC7A11 inhibits cell ferroptosis in osteosarcoma. Biochem. Biophys. Res. Commun..

[136] He P., Liu F., Wang Z., Gong H., Zhang M., Jia Z., Zhai X. (2022). CircKIF4A enhances osteosarcoma proliferation and metastasis by sponging MiR-515-5p and upregulating SLC7A11. Mol. Biol. Rep..

[137] Li M.J., Li F., Xu J., Liu Y.D., Hu T., Chen J.T. (2016). rhHMGB1 drives osteoblast migration in a TLR2/TLR4- and NF-κB-dependent manner. Biosci. Rep..

[138] Zhu H., Dai R., Zhou Y., Fu H., Meng Q. (2018). TLR2 Ligand Pam3CSK4 Regulates MMP-2/9 Expression by MAPK/NF-κB Signaling Pathways in Primary Brain Microvascular Endothelial Cells. Neurochem. Res..

[139] Ozerdem U. (2006). Targeting pericytes diminishes neovascularization in orthotopic uveal melanoma in nerve/glial antigen 2 proteoglycan knockout mouse. Ophthalmic Res..

[140] Sato S., Tang Y.J., Wei Q., Hirata M., Weng A., Han I., Okawa A., Takeda S., Whetstone H., Nadesan P. (2016). Mesenchymal Tumors Can Derive from Ng2/Cspg4-Expressing Pericytes with β-Catenin Modulating the Neoplastic Phenotype. Cell Rep..

[141] Zhao Z.X., Li X., Liu W.D., Liu X.Z., Wu S.J., Hu X.H. (2016). Inhibition of Growth and Metastasis of Tumor in Nude Mice after Intraperitoneal Injection of Bevacizumab. Orthop. Surg..

[142] Navid F., Santana V.M., Neel M., McCarville M.B., Shulkin B.L., Wu J., Billups C.A., Mao S., Daryani V.M., Stewart C.F. (2017). A phase II trial evaluating the feasibility of adding bevacizumab to standard osteosarcoma therapy. Int. J. Cancer.

[143] Volz N.B., Stintzing S., Zhang W., Yang D., Ning Y., Wakatsuki T., El-Khoueiry R.E., Li J.E., Kardosh A., Loupakis F. (2015). Genes involved in pericyte-driven tumor maturation predict treatment benefit of first-line FOLFIRI plus bevacizumab in patients with metastatic colorectal cancer. Pharmacogenom. J..

[144] Chen D., Fan Z., Rauh M., Buchfelder M., Eyupoglu I.Y., Savaskan N. (2017). ATF4 promotes angiogenesis and neuronal cell death and confers ferroptosis in a xCT-dependent manner. Oncogene.

[145] Briggs K.J., Koivunen P., Cao S., Backus K.M., Olenchock B.A., Patel H., Zhang Q., Signoretti S., Gerfen G.J., Richardson A.L. (2016). Paracrine Induction of HIF by Glutamate in Breast Cancer: EglN1 Senses Cysteine. Cell.

[146] Wang Z., Zong H., Liu W., Lin W., Sun A., Ding Z., Chen X., Wan X., Liu Y., Hu Z. (2024). Augmented ERO1α upon mTORC1 activation induces ferroptosis resistance and tumor progression via upregulation of SLC7A11. J. Exp. Clin. Cancer Res..

[147] West X.Z., Malinin N.L., Merkulova A.A., Tischenko M., Kerr B.A., Borden E.C., Podrez E.A., Salomon R.G., Byzova T.V. (2010). Oxidative stress induces angiogenesis by activating TLR2 with novel endogenous ligands. Nature.

[148] Giri S., Lamichhane G., Pandey J., Khadayat R., K C.S., Devkota H.P., Khadka D. (2025). Immune Modulation and Immunotherapy in Solid Tumors: Mechanisms of Resistance and Potential Therapeutic Strategies. Int. J. Mol. Sci..

[149] Thanindratarn P., Dean D.C., Nelson S.D., Hornicek F.J., Duan Z. (2019). Advances in immune checkpoint inhibitors for bone sarcoma therapy. J. Bone Oncol..

[150] Koopmans I., Hendriks M., van Ginkel R.J., Samplonius D.F., Bremer E., Helfrich W. (2019). Bispecific Antibody Approach for Improved Melanoma-Selective PD-L1 Immune Checkpoint Blockade. J. Investig. Dermatol..

[151] Boudin L., de Nonneville A., Finetti P., Mescam L., Le Cesne A., Italiano A., Blay J.Y., Birnbaum D., Mamessier E., Bertucci F. (2022). CSPG4 expression in soft tissue sarcomas is associated with poor prognosis and low cytotoxic immune response. J. Transl. Med..

[152] Wang L., Zhang Q., Chen W., Shan B., Ding Y., Zhang G., Cao N., Liu L., Zhang Y. (2013). B7-H3 is overexpressed in patients suffering osteosarcoma and associated with tumor aggressiveness and metastasis. PLoS ONE.

[153] Zhang Q., Zhang Z., Liu G., Li D., Gu Z., Zhang L., Pan Y., Cui X., Wang L., Liu G. (2022). B7-H3 targeted CAR-T cells show highly efficient anti-tumor function against osteosarcoma both in vitro and in vivo. BMC Cancer.

[154] McEachron T.A., Triche T.J., Sorenson L., Parham D.M., Carpten J.D. (2018). Profiling targetable immune checkpoints in osteosarcoma. Oncoimmunology.

[155] Majzner R.G., Theruvath J.L., Nellan A., Heitzeneder S., Cui Y., Mount C.W., Rietberg S.P., Linde M.H., Xu P., Rota C. (2019). CAR T Cells Targeting B7-H3, a Pan-Cancer Antigen, Demonstrate Potent Preclinical Activity Against Pediatric Solid Tumors and Brain Tumors. Clin. Cancer Res..

[156] Cascini C., Ratti C., Botti L., Parma B., Cancila V., Salvaggio A., Meazza C., Tripodo C., Colombo M.P., Chiodoni C. (2023). Rewiring innate and adaptive immunity with TLR9 agonist to treat osteosarcoma. J. Exp. Clin. Cancer Res..

[157] Oyama R., Nabeshima A., Endo M., Novikov A., Fujiwara T., Phelip C., Yokoyama N., Oda Y., Caroff M., Matsumoto Y. (2025). A detoxified TLR4 agonist inhibits tumour growth and lung metastasis of osteosarcoma by promoting CD8+ cytotoxic lymphocyte infiltration. BJC Rep..

[158] Sutmuller R.P., den Brok M.H., Kramer M., Bennink E.J., Toonen L.W., Kullberg B.J., Joosten L.A., Akira S., Netea M.G., Adema G.J. (2006). Toll-like receptor 2 controls expansion and function of regulatory T cells. J. Clin. Investig..

[159] McBride A., Konowich J., Salgame P. (2013). Host defense and recruitment of Foxp3^+^ T regulatory cells to the lungs in chronic Mycobacterium tuberculosis infection requires toll-like receptor 2. PLoS Pathog..

[160] Ye L., Zhang Q., Cheng Y., Chen X., Wang G., Shi M., Zhang T., Cao Y., Pan H., Zhang L. (2018). Tumor-derived exosomal HMGB1 fosters hepatocellular carcinoma immune evasion by promoting TIM-1^+^ regulatory B cell expansion. J. Immunother. Cancer.

[161] Chen Y.Q., Li P.C., Pan N., Gao R., Wen Z.F., Zhang T.Y., Huang F., Wu F.Y., Ou X.L., Zhang J.P. (2019). Tumor-released autophagosomes induces CD4^+^ T cell-mediated immunosuppression via a TLR2–IL-6 cascade. J. Immunother. Cancer.

[162] Wang Z., Yang C., Li L., Jin X., Zhang Z., Zheng H., Pan J., Shi L., Jiang Z., Su K. (2020). Tumor-derived HMGB1 induces CD62L^dim^ neutrophil polarization and promotes lung metastasis in triple-negative breast cancer. Oncogenesis.

[163] Niu X., Yin L., Yang X., Yang Y., Gu Y., Sun Y., Yang M., Wang Y., Zhang Q., Ji H. (2022). Serum amyloid A 1 induces suppressive neutrophils through the Toll-like receptor 2-mediated signaling pathway to promote progression of breast cancer. Cancer Sci..

[164] Flores R.J., Kelly A.J., Li Y., Chen X., McGee C., Krailo M., Barkauskas D.A., Hicks J., Man T.K. (2017). The prognostic significance of circulating serum amyloid A and CXC chemokine ligand 4 in osteosarcoma. Pediatr. Blood Cancer.

[165] Jin J., Byun J.K., Choi Y.K., Park K.G. (2023). Targeting glutamine metabolism as a therapeutic strategy for cancer. Exp. Mol. Med..

[166] Ren L., Ruiz-Rodado V., Dowdy T., Huang S., Issaq S.H., Beck J., Wang H., Tran Hoang C., Lita A., Larion M. (2020). Glutaminase-1 (GLS1) inhibition limits metastatic progression in osteosarcoma. Cancer Metab..

[167] Tang H.Y., Guo J.Q., Sang B.T., Cheng J.N., Wu X.M. (2022). PDGFRβ modulates aerobic glycolysis in osteosarcoma HOS cells via the PI3K/AKT/mTOR/c-Myc pathway. Biochem. Cell Biol..

[168] Sheng G., Gao Y., Ding Q., Zhang R., Wang T., Jing S., Zhao H., Ma T., Wu H., Yang Y. (2023). P2RX7 promotes osteosarcoma progression and glucose metabolism by enhancing c-Myc stabilization. J. Transl. Med..

[169] Koppula P., Zhang Y., Shi J., Li W., Gan B. (2017). The glutamate/cystine antiporter SLC7A11/xCT enhances cancer cell dependency on glucose by exporting glutamate. J. Biol. Chem..

[170] Shin C.S., Mishra P., Watrous J.D., Carelli V., D’Aurelio M., Jain M., Chan D.C. (2017). The glutamate/cystine xCT antiporter antagonizes glutamine metabolism and reduces nutrient flexibility. Nat. Commun..

[171] Song Z., Yao Q., Huang L., Cui D., Xie J., Wu L., Huang J., Zhai B., Liu D., Xu X. (2025). Glucose Deprivation-Induced Disulfidptosis via the SLC7A11-INF2 Axis: Pan-Cancer Prognostic Exploration and Therapeutic Validation. Adv. Sci..

[172] Wan Z., Sun R., Liu Y.W., Li S., Sun J., Li J., Zhu J., Moharil P., Zhang B., Ren P. (2021). Targeting metabotropic glutamate receptor 4 for cancer immunotherapy. Sci. Adv..

[173] de Aquino M.T.P., Hodo T.W., Ochoa S.G., Uzhachenko R.V., Mohammed M.A., Goodwin J.S., Kanagasabai T., Ivanova A.V., Shanker A. (2025). Glutamate receptor-T cell receptor signaling potentiates full CD8^+^ T cell activation and effector function in tumor immunity. iScience.

[174] Srivastava M.K., Sinha P., Clements V.K., Rodriguez P., Ostrand-Rosenberg S. (2010). Myeloid-derived suppressor cells inhibit T-cell activation by depleting cystine and cysteine. Cancer Res..

[175] Han C., Ge M., Xing P., Xia T., Zhang C., Ma K., Ma Y., Li S., Li W., Liu X. (2024). Cystine deprivation triggers CD36-mediated ferroptosis and dysfunction of tumor infiltrating CD8^+^ T cells. Cell Death Dis..

[176] Long Y., Tao H., Karachi A., Grippin A.J., Jin L., Chang Y.E., Zhang W., Dyson K.A., Hou A.Y., Na M. (2020). Dysregulation of Glutamate Transport Enhances Treg Function That Promotes VEGF Blockade Resistance in Glioblastoma. Cancer Res..

[177] He Q., Liu M., Huang W., Chen X., Zhang B., Zhang T., Wang Y., Liu D., Xie M., Ji X. (2021). IL-1β-Induced Elevation of Solute Carrier Family 7 Member 11 Promotes Hepatocellular Carcinoma Metastasis Through Up-regulating Programmed Death Ligand 1 and Colony-Stimulating Factor 1. Hepatology.

[178] Tang B., Zhu J., Wang Y., Chen W., Fang S., Mao W., Xu Z., Yang Y., Weng Q., Zhao Z. (2023). Targeted xCT-mediated Ferroptosis and Protumoral Polarization of Macrophages Is Effective against HCC and Enhances the Efficacy of the Anti-PD-1/L1 Response. Adv. Sci..

[179] Arensman M.D., Yang X.S., Leahy D.M., Toral-Barza L., Mileski M., Rosfjord E.C., Wang F., Deng S., Myers J.S., Abraham R.T. (2019). Cystine-glutamate antiporter xCT deficiency suppresses tumor growth while preserving antitumor immunity. Proc. Natl. Acad. Sci. USA.

[180] Giraudo L., Cattaneo G., Gammaitoni L., Iaia I., Donini C., Massa A., Centomo M.L., Basirico M., Vigna E., Pisacane A. (2023). CSPG4 CAR-redirected Cytokine Induced Killer lymphocytes (CIK) as effective cellular immunotherapy for HLA class I defective melanoma. J. Exp. Clin. Cancer Res..

[181] Xiong Q., Yin B., Jiang H., Qiu Y., Shi G., Xu J., Xu T., Deng H. (2025). Targeting CSPG4 enhances the anti-tumor activity of CAR-NK cells for glioblastoma. Cell. Oncol..

[182] Leuci V., Donini C., Grignani G., Rotolo R., Mesiano G., Fiorino E., Gammaitoni L., D’Ambrosio L., Merlini A., Landoni E. (2020). CSPG4-Specific CAR.CIK Lymphocytes as a Novel Therapy for the Treatment of Multiple Soft-Tissue Sarcoma Histotypes. Clin. Cancer Res..

[183] Chauhan J., Grandits M., Palhares L., Mele S., Nakamura M., Lopez-Abente J., Crescioli S., Laddach R., Romero-Clavijo P., Cheung A. (2023). Anti-cancer pro-inflammatory effects of an IgE antibody targeting the melanoma-associated antigen chondroitin sulfate proteoglycan 4. Nat. Commun..

[184] Hoffmann R.M., Crescioli S., Mele S., Sachouli E., Cheung A., Chui C.K., Andriollo P., Jackson P.J.M., Lacy K.E., Spicer J.F. (2020). A Novel Antibody-Drug Conjugate (ADC) Delivering a DNA Mono-Alkylating Payload to Chondroitin Sulfate Proteoglycan (CSPG4)-Expressing Melanoma. Cancers.

[185] de Bruyn M., Rybczynska A.A., Wei Y., Schwenkert M., Fey G.H., Dierckx R.A., van Waarde A., Helfrich W., Bremer E. (2010). Melanoma-associated Chondroitin Sulfate Proteoglycan (MCSP)-targeted delivery of soluble TRAIL potently inhibits melanoma outgrowth in vitro and in vivo. Mol. Cancer.

[186] Yu L., Favoino E., Wang Y., Ma Y., Deng X., Wang X. (2011). The CSPG4-specific monoclonal antibody enhances and prolongs the effects of the BRAF inhibitor in melanoma cells. Immunol. Res..

[187] Greiner D., Xue Q., Waddell T.Q., Kurudza E., Chaudhary P., Belote R.L., Dotti G., Judson-Torres R.L., Reeves M.Q., Cheshier S.H. (2025). Human CSPG4-targeting CAR-macrophages inhibit melanoma growth. Oncogene.

[188] Cattaneo G., Ventin M., Arya S., Bailey C., Vantaku V.R., Jia J., Qi M., Maggs L., Wang X., Parangi S. (2025). B7-H3 and CSPG4-targeted CAR T cells as potent effectors in anaplastic thyroid cancer. J. Exp. Clin. Cancer Res..

[189] Mittelman A., Chen G.Z., Wong G.Y., Liu C., Hirai S., Ferrone S. (1995). Human high molecular weight-melanoma associated antigen mimicry by mouse anti-idiotypic monoclonal antibody MK2-23: Modulation of the immunogenicity in patients with malignant melanoma. Clin. Cancer Res..

[190] Vincze O., Spada B., Bilder D., Cagan A., DeGregori J., Gorbunova V., Maley C.C., Schiffman J.D., Seluanov A., Giraudeau M. (2025). Advancing cancer research via comparative oncology. Nat. Rev. Cancer.

[191] Ruzzi F., Riccardo F., Conti L., Tarone L., Semprini M.S., Bolli E., Barutello G., Quaglino E., Lollini P.L., Cavallo F. (2025). Cancer vaccines: Target antigens, vaccine platforms and preclinical models. Mol. Asp. Med.

[192] Roerden M., Spranger S. (2025). Cancer immune evasion, immunoediting and intratumour heterogeneity. Nat. Rev. Immunol..

[193] Thanee M., Padthaisong S., Suksawat M., Dokduang H., Phetcharaburanin J., Klanrit P., Titapun A., Namwat N., Wangwiwatsin A., Sa-Ngiamwibool P. (2021). Sulfasalazine modifies metabolic profiles and enhances cisplatin chemosensitivity on cholangiocarcinoma cells in in vitro and in vivo models. Cancer Metab..

[194] Dixon S.J., Patel D.N., Welsch M., Skouta R., Lee E.D., Hayano M., Thomas A.G., Gleason C.E., Tatonetti N.P., Slusher B.S. (2014). Pharmacological inhibition of cystine-glutamate exchange induces endoplasmic reticulum stress and ferroptosis. eLife.

[195] Dolma S., Lessnick S.L., Hahn W.C., Stockwell B.R. (2003). Identification of genotype-selective antitumor agents using synthetic lethal chemical screening in engineered human tumor cells. Cancer Cell.

[196] Wilhelm S., Carter C., Lynch M., Lowinger T., Dumas J., Smith R.A., Schwartz B., Simantov R., Kelley S. (2006). Discovery and development of sorafenib: A multikinase inhibitor for treating cancer. Nat. Rev. Drug Discov..

[197] Li Y., Yan H., Xu X., Liu H., Wu C., Zhao L. (2020). Erastin/sorafenib induces cisplatin-resistant non-small cell lung cancer cell ferroptosis through inhibition of the Nrf2/xCT pathway. Oncol. Lett..

[198] Zhang R., Thoroe-Boveleth S., Chigrin D.N., Kiessling F., Lammers T., Pallares R.M. (2024). Nanoscale engineering of gold nanostars for enhanced photoacoustic imaging. J. Nanobiotechnol..

[199] Huang L., Ge X., Liu Y., Li H., Zhang Z. (2022). The Role of Toll-like Receptor Agonists and Their Nanomedicines for Tumor Immunotherapy. Pharmaceutics.

[200] Quaglino E., Conti L., Cavallo F. (2020). Breast cancer stem cell antigens as targets for immunotherapy. Semin. Immunol..

[201] Tye H., Kennedy C.L., Najdovska M., McLeod L., McCormack W., Hughes N., Dev A., Sievert W., Ooi C.H., Ishikawa T.O. (2012). STAT3-driven upregulation of TLR2 promotes gastric tumorigenesis independent of tumor inflammation. Cancer Cell.

